# Developing gene-tagged molecular markers for evaluation of genetic association of apple *SWEET* genes with fruit sugar accumulation

**DOI:** 10.1038/s41438-018-0024-3

**Published:** 2018-03-20

**Authors:** Qiaoling Zhen, Ting Fang, Qian Peng, Liao Liao, Li Zhao, Albert Owiti, Yuepeng Han

**Affiliations:** 1 Key Laboratory of Plant Germplasm Enhancement and Specialty Agriculture, Wuhan Botanical Garden of the Chinese Academy of Sciences, Wuhan, 430074 China; 2 Graduate University of Chinese Academy of Sciences, 19A Yuquanlu, Beijing, 100049 China; 3 Sino-African Joint Research Center, Chinese Academy of Sciences, Wuhan, 430074 China; 4 College of Horticulture Science and Engineering, Shandong Agricultural University, Tai-An, Shandong 271018 China

## Abstract

Sugar content is an important component of fruit quality. Although sugar transporters are known to be crucial for sugar accumulation, the role of genes encoding SWEET sugar transporters in fruit sugar accumulation remains elusive. Here we report the effect of the *SWEET* genes on fruit sugar accumulation in apple. A total of 25 *MdSWEET* genes were identified in the apple genome, and 9 were highly expressed throughout fruit development. Molecular markers of these 9 *MdSWEET* genes were developed and used for genotyping of 188 apple cultivars. The association of polymorphic *MdSWEET* genes with soluble sugar content in mature fruit was analyzed. Three genes, *MdSWEET2e*, *MdSWEET9b*, and *MdSWEET15a*, were significantly associated with fruit sugar content, with *MdSWEET15a* and *MdSWEET9b* accounting for a relatively large proportion of phenotypic variation in sugar content. Moreover, both *MdSWEET9b* and *MdSWEET15a* are located on chromosomal regions harboring QTLs for sugar content. Hence, *MdSWEET9b* and *MdSWEET15a* are likely candidates regulating fruit sugar accumulation in apple. Our study not only presents an efficient way of implementing gene functional study but also provides molecular tools for genetic improvement of fruit quality in apple-breeding programs.

## Sugar transportation: uncovering fruit sugar regulation in apples

Insights into the mechanisms that drive fruit sugar content in apples could help develop and breed apple trees with improved fruit quality. The transportation of sugars throughout fruit plants plays a vital role both in healthy plant development and in the quality of the fruit produced. However, the mechanisms inherent in fruit sugar accumulation are not yet fully understood. Yuepeng Han at the Chinese Academy of Sciences in Wuhan, China, and co-workers sought to clarify the role of genes encoding *SWEET* sugar transporters in the cultivated apple, *Malus x domestica* Borkh. They identified 25 *SWEET* genes in the apple genome, with 9 of these highly expressed during fruit development. Han’s team narrowed these genes down to two that were highly associated with the regulation of sugar content in the fruit.

## INTRODUCTION

Sugar is the main carbon source and energy-supplying substance in organisms, and it plays an important role in plant growth and development. Sugar is the main product of photosynthesis and carbon dioxide assimilation occurs mainly in stromal cells of chloroplast. The main assimilation product synthesized in chloroplasts is triose phosphate, most of which will be converted to either sucrose in the cytosol or starch in the chloroplast^1,2^. Sucrose is commonly translocated to other carbon-demanding organs through the long-distance transport occurring in phloem. Hence, sugar transportation is critical for maintaining the source-sink balance^3^.

Over the past two decades, various sugar transporters have been identified in living organisms, including plants, animals, humans, and fungi^4–6^. These transporters can be categorized into four families: sodium solute symporter transporters; major facilitator superfamily transporters; phosphotransferase system transporters; and sugar will eventually be exported transporters (*SWEET*s). Among these transporters, *SWEET*s emerge as a unique and novel class of sugar transporters. *SWEET*s are conserved evolutionarily and exist widely in eukaryotes, and prokaryotes such as arechaea and eubacteria. The first member of the *SWEET* gene family, *MtN3*, was identified in *Medicago truncatula*, and its expression was induced during root nodule development^7^. Later, a homolog of the *MtN3* gene, designated Saliva, was identified in Drosophila and displayed salivary gland-specific expression during embryonic development^8^. Recently, MtN3/Saliva-type genes were functionally characterized as sugar transporters in both animals and plants, and thus gave the name “*SWEET*”^9^. SWEET proteins are characterized by the MtN3/Saliva motif (also known as the PQ-loop repeat), which comprises three alpha-helical transmembrane domains (3-TMs). Eukaryotic *SWEET*s consists of a tandem repeat of the basic 3-TM unit separated by a single transmembrane domain, which constitutes a 3-1-3 TM structure. In contrast, prokaryotic *SWEET*s, also called *SemiSWEET*s, contain only a single 3-TM unit, suggesting that eukaryotic *SWEET*s evolved through a duplication of the 3-TM unit^10^.

In plants, *SWEET*s function as bidirectional uniporters that mediate influx and efflux of sugars across cell membranes. *SWEET*s can be divided into four clades^11^. Clades I, II, and IV *SWEET*s transport predominantly hexoses, whilst clade III *SWEET*s appear to be sucrose transporters (SUTs)^12–15^. For example, the clade I *SWEET AtSWEET*1 and the clade II *SWEET OsSWEET*5 mediate the uptake and efflux of glucose or galactose, respectively, across the cell membrane^9,16^. Two clade III *SWEET*s, *AtSWEET11* and *AtSWEET12*, are involved in efflux of photosynthesized sucrose from phloem parenchyma cells into intercellular space for phloem loading and long-distance translocation of sucrose^17^. A newly identified clade III *SWEET*, *AtSWEET9*, transports sucrose to the apoplast and plays an essential role in nectar secretion^18^. The clade IV *SWEET*, *AtSWEET17*, functions as a fructose-specific uniporter, with a key role in facilitating bidirectional transport of fructose across the tonoplast in leaf and root^12,13^. In addition, *SWEET*s have been shown to affect various physiological processes, such as pollen development^19,20^, seed filling^21,22^, stress and senescence^14,16,23,24^, modulating gibberellins response^25^, and host–pathogen interaction^26^. Usually, abiotic stress such as cold, high salinity, and drought, and biotic stress caused by fungi or bacteria result in an induction of specific *SWEET* genes^27,28^.

The accumulation of carbohydrates in storage organs such as seeds and fruits mainly depends on the supply of photoassimilates from photosynthetic tissues, especially source leaves. In most plants, sucrose is the major carbohydrate transported over a long distance in the veins to support the growth and development of storage organs^6^. Sugar transporter genes exhibit divergent evolutionary patterns and play important roles in sugar accumulation in plants^29^. For example, genes encoding SUT proteins are involved in sugar translocation toward storage organs^30–32^. Recently, the *SWEET4* gene is also found to play an important role in seed filling^22^. In maize and rice, *SWEET4* shows high expression during seed development and contributes to seed filling by enhancing the importation of hexoses into the endosperm. This study sheds light on our understanding on the mechanism by which sucrose is released from maternal tissues such as seed coat to support filial tissues such as embryonic tissue. Since sugar content is an important component of fruit quality, increasing attention has also been paid to investigate the *SWEET* gene family in fruit crops such as apple^33^, grapevine^34^, and banana^35^. However, the effect of *SWEET*s on fruit sugar accumulation remains elusive.

The domesticated apple, *Malus x domestica* Borkh., is an economically important fruit crop worldwide. Apple belongs to the family Rosaceae, and the cultivated apple is a diploidized autopolyploid species with a basic chromosome number of *x* = 17. The draft genome sequence of the domesticated apple has been released, and accounts for an approximately 750 Mb per haploid^36^. In this study, we report the identification of the *MdSWEET* genes with high expression during apple fruit development. DNA markers for these *MdSWEET*s were developed to investigate their association with fruit sugar content. Our study not only aids our better understanding of the effect of *SWEET*s on fruit sugar accumulation but it will also be helpful for genetic improvement of fruit sugar accumulation in apple-breeding programs.

## MATERIALS AND METHODS

### Plant material

All 188 apple cultivars (Table S1) used in this study that show a great variation in fruit sweetness are maintained at Xingcheng Institute of Pomology of the Chinese Academy of Agricultural Sciences, Xingcheng, Liaoning, China. Young leaves used for genomic DNA extraction were collected in the spring of 2015. Leaf samples were immediately frozen in liquid nitrogen, and then stored at −80 °C until use. Fruits at mature stage were randomly collected in 2015 and fruit maturity was comprehensively estimated based on skin background color and blush development, seed color turning into brown, and the previous records of maturity date. Each cultivar had three replicates, consisting of nine fruits. Fruit samples were cut into small pieces, immediately frozen in liquid nitrogen, and then stored either at −40 °C for sugar measurement or at −80 °C for real-time PCR (RT-PCR) analysis.

### Measurement of soluble sugar content

The content of soluble sugar components was measured using high-performance liquid chromatography (HPLC) according to our previous report^37^. Briefly, fruit samples were ground into fine powder in liquid nitrogen using an A11 basic Analytical mill (IKA, Germany). One gram of powder was dissolved in 6 ml sterilized deionized water, the mixture was extracted in an ultrasonic bath for 30 min, and then centrifuged at 6000 r/s for 15 min. The supernatant was collected, purified using a SEPC18 syringe (Supelclean ENVI C18 SPE), and subsequently filtered through a 0.22 μm Sep-Pak filter. The filtered supernatant was used for sugar content measurement using a Dionex P680 HPLC system (Dionex Corporation, CA, USA) equipped with a refractive index detector (Shodex RI-101; Shodex Munich, Germany). The separation was performed on a Transgenomic COREGET-87C column (7.8 mm × 300 mm, 10 μm) together with a guard column Transgenomic CARB Sep Coregel 87C. The column temperature was maintained at 85 °C by using a Dionex TCC-100 thermostated column compartment. The mobile phase was set at a flow rate of 0.6 ml/min with degassed, distilled, deionized water. Peak areas were integrated with the Chromeleon chromatography data system according to external standard solution calibrations (reagents from Sigma Chemical Co., Castle Hill, NSW, Australia). Sugar concentrations were expressed on a fresh weight (FW) basis, and total sugar content was indicated by the amount of four sugars found in apple fruit, i.e., glucose, sucrose, fructose, and sorbitol. In addition, the measurement of soluble solid content (SSC) was conducted using a pocket refractometer (Atago, Tokyo, Japan).

### Identification of the *SWEET* genes in apple and their phylogenetic analysis

Coding DNA sequences of the *SWEET* gene family in *Arabidopsis thaliana* were retrieved from the Arabidopsis Information Resource (TAIR, http://www.arabidopsis.org/). These coding sequences were used as query sequences to compare against the apple genome sequence database (GDDH13 V1.1, https://www.rosaceae.org/blast/) by BlastX with a cutoff *E*-value of 1.00E-10. The homologs of *Arabidopsis SWEET* genes in the apple genome were named according to their phylogenetic relationships to the founding members of the family in *Arabidopsis*^9^. Chromosome lengths and gene locations were presented according to the draft genome sequence of the doubled haploid (GDDH13 V1.1)^38^.

Multiple alignment of amino-acid sequences of *SWEET* genes in *Arabidopsis* and apple was conducted using the integrated MUSCLE alignment program in MEGA5 (Molecular Evolutionary GenetiMd Analysis) with default parameters^39^. The resulting data matrix was analyzed using the Neighbor-Joining method. The bootstrap consensus tree was inferred from 1000 replicates and the bootstrap values <50% were collapsed.

### RNA isolation and quantitative RT-PCR

Two apple cultivars, K9 and Shizishan 2, were randomly selected for quantitative RT-PCR (qRT-PCR) analysis. Fruit samples were collected at 30, 60, and 90 days after full bloom. Each cultivar had three biological replicates, containing of nine fruits. Fruits of each replicate were cut into small pieces, mixed, and used for total RNA extraction. RNA extraction was conducted using RNA prep Pure Plant Kit (TianGen, Beijing, China) according to the manufacturer’s instructions. RNA concentration and quantity were detected and assessed with NanoDrop2000 (Thermo Scientific).

Approximately 1 µg of total RNA was used to synthesize the first strand of cDNA using TransScript One-Step gDNA Removal and cDNA Synthesis SuperMix (TRANS, Beijing, China) following the manufacturer’s instructions. qRT-PCR was performed in 20 µL reaction containing 1× SYBR Green II Master Mix (Takara, Dalian, China), 0.2 µM of each primer, and 0.5 µL of template cDNA. The qRT-PCR amplifications were performed using the Applied Biosystems 7500 Real-Time PCR System (Applied Biosystems, USA), and the reaction program was set as follows: 95 °C for 1 min, one cycle; and 95 °C for 5 s, 60 °C for 34 s, 40 cycles. Melting curve analysis was performed at the end of 40 cycles by heating from 55 to 95 °C at a rate of 0.5 °C/s. An apple polymer ubiquitin enzyme gene (*UBQ*) was selected as a constitute control^40^. The relative expression level of all detected genes was calculated according to the cycle threshold 2^−ΔΔCT^ method. All analyses were performed in triplicates. The primer sequences are listed in Table S2.

### Development of gene-tagged simple sequence repeat and cleaved amplified polymorphism sequence markers

Simple sequence repeat (SSR) or cleaved amplified polymorphism sequence (CAPS) markers were development for *SWEET*s that showed high expression in fruit of apple. SSR Hunter software and dCAPS Finder 2.0 (http://helix.wustl.edu/dcaps/dcaps.html) were used to screen SSRs with ≥7 repeats or CAPS markers, respectively, in genomic DNA sequences of each *SWEET* gene, including 2 kb upstream of the start codon, the entire coding region, and 2 kb downstream of the translation stop codon. Primers flanking the SSR and CAPS loci were designed using the Primer 5 program, and polymorphism of the SSR and CAPS markers was evaluated using four apple cultivars with high sugar content and four cultivars with low sugar content.

PCR amplification was performed using the GeneAmp PCR System 9700 (ABI, USA) with the following condition: 3 min at 94 °C, followed by 35 cycles consisting of 94 °C for 30 s, 60 °C for 30 s, 72 °C for 30 s, and with a final extension of 72 °C for 7 min. For the SSR markers, 3 μL of amplification products was mixed with an equal volume of formamide loading buffer (98 % formamide, 10 mM EDTA, pH 8.0, 0.025% bromophenol blue, and xylene cyanol). The mixture was denatured at 95 °C for 5 min, and then immediately put on ice for 5 min. An aliquot of 2 μL mixture was on an 8% polyacrylamide gel and electrophoresed for 1–1.5 h at 1000 V. DNA bands were visualized after silver staining, and their sizes were estimated on the basis of a standard 25 bp DNA ladder. For the CAPS markers, amplification products were digested with restriction enzymes and then separated on 2% agarose gel. DNA bands in agrose gel were visualized under ultraviolet light after staining with ethidium bromide.

### Statistical analysis

A total of 188 apple cultivars were subjected to statistical analysis. Each cultivar was genotyped for the *SWEET* loci following analysis of molecular marker profiles. The detection of association between molecular markers and fruit sugar accumulation was performed with the software package TASSEL version 3.0 according to our previous report^41^. The criterion for marker–trait association was set at *P* ≤ 0.01. Fisher’s least significant difference at *P* < 0.01 was used to compare mean soluble sugar contents between cultivars.

## RESULTS

### The *SWEET*s gene family in the apple genome

**Fig. 1:**
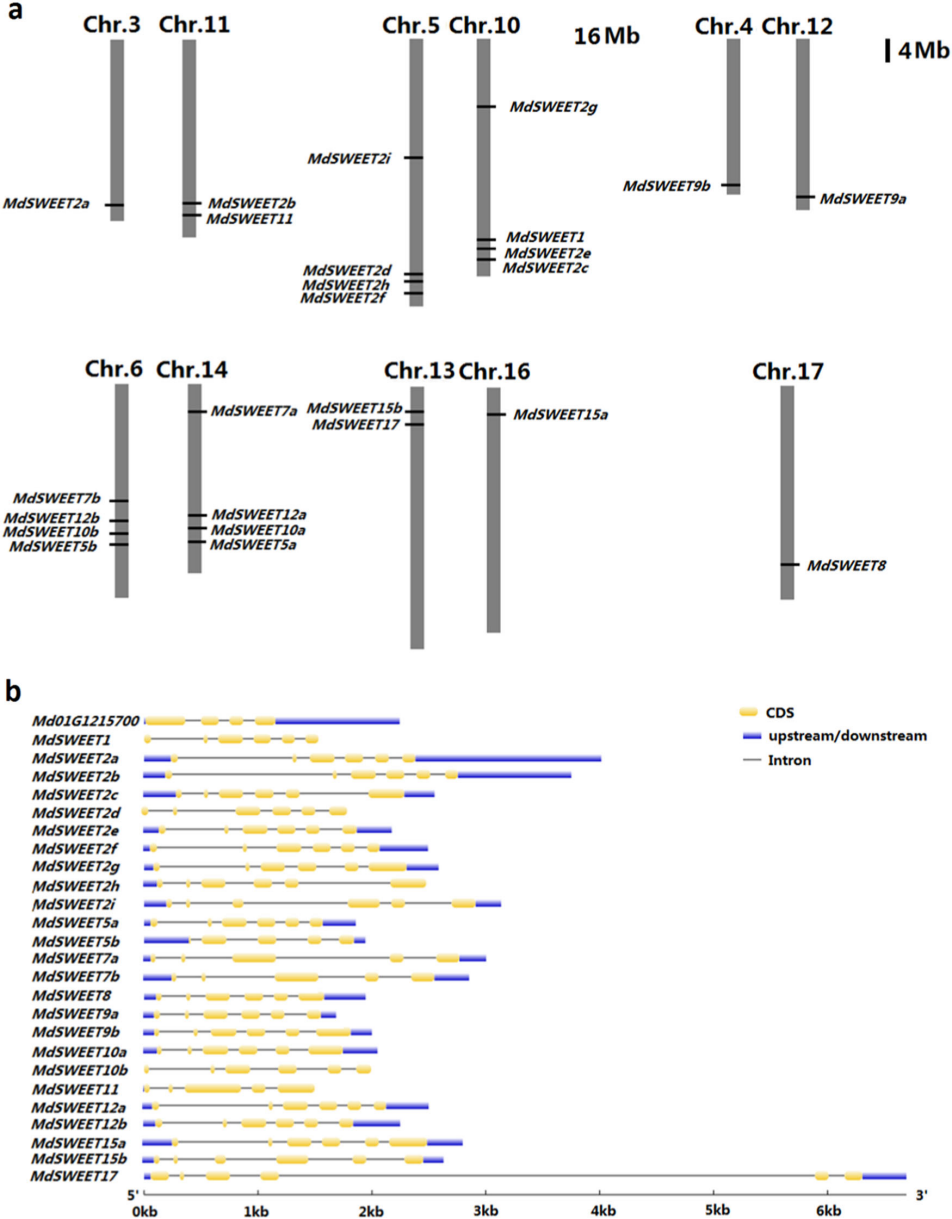
Chromosomal location of the *SWEET* gene family in apple (**a**) and their genomic structure (**b**)

A total of 25 *MdSWEET* genes were identified in the apple genome. Of the 25 *MdSWEET* genes, 24 were located on five homologous pairs of chromosomes (3–11, 5–10, 4–12, 6–14, 13–16), and 1 on chromosome 17 (Fig. 1a). Genomic structural analysis showed that the majority of *MdSWEET* genes consisted of six exons, while 4 *MdSWEET* genes, *MdSWEET5b*, *MdSWEET7a*, *MdSWEET7b*, and *MdSWEET11*, contained five exons (Fig. 1b and Table S3). The open reading frames of the *MdSWEET* genes ranged from 645 to 1020 bp in length and their deduced proteins ranging from 215 to 340 amino acids in length (Table S3). The conserved domain prediction indicated that 21 *MdSWEET* genes had seven alpha-helical TMs. By contrast, 3 *MdSWEET* genes, *MdSWEET5a*, *MdSWEET9a*, and *MdSWEET5b*, had six TMs, with absence of the TM7 domain (Table S3). Interestingly, the remaining *MdSWEET11* gene had eight TMs. In addition, it is worth noting that an additional *SWEET* gene (GDR accession no. MD01G1215700) was also found in the apple genome. This *SWEET* gene was located on chromosome 1, with four exons (Fig. 1b), contained only one MtN3/Saliva motif, and showed extremely low expression throughout fruit development. Hence, this *SWEET* gene was deemed a pseudogene and was not included in the later analysis.

**Fig. 2:**
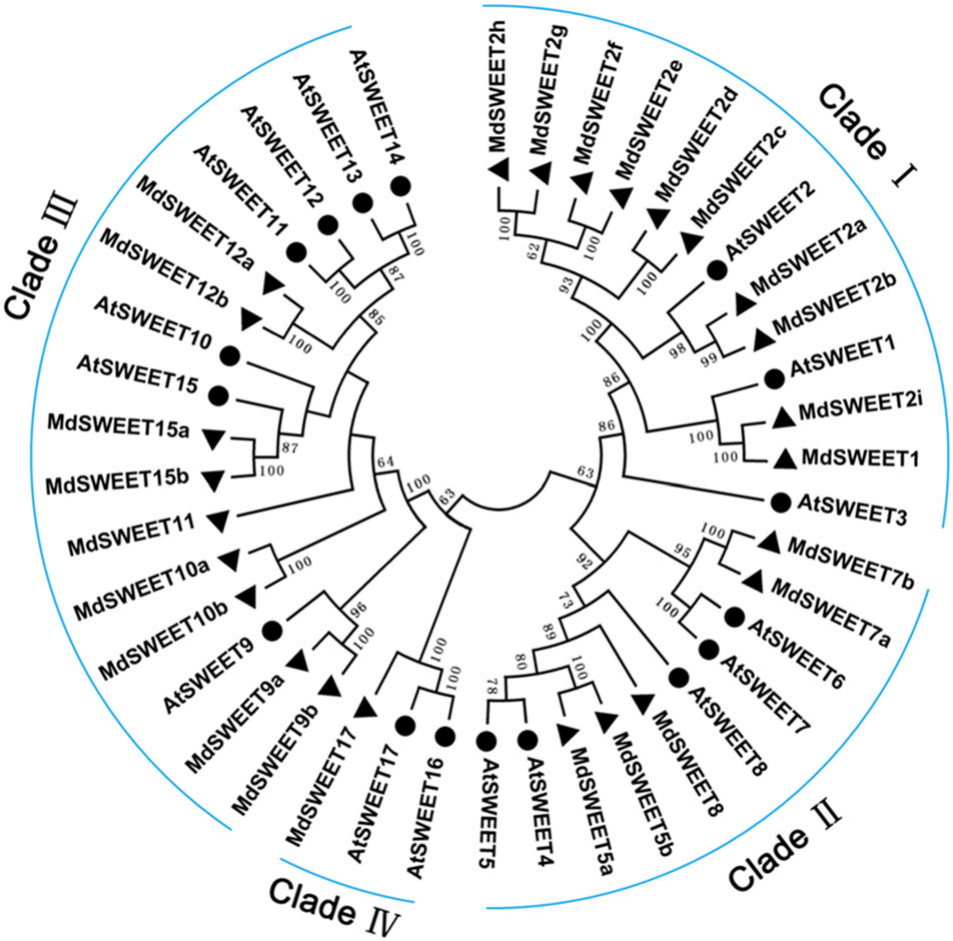
Phylogenetic tree derived from the amino-acid sequences of 25 *MdSWEET* genes in apple and 17 *AtSWEET* genes in *Arabidopsis*. All the *SWEET* genes are divided into four clades (I–IV), and numbers near branches represent bootstrap values. The circles and triangles indicate SWEETs from *Arabidopsis* and apple, respectively

Phylogenetic analysis revealed that all the *MdSWEET* genes were divided into four clades as previously defined by Chen et al.^9^, and each *MdSWEET* gene was named following its homologous genes in *Arabidopsis* (Fig. 2). Clade I, II, III, and IV contained 10, 5, 9, and 1 *MdSWEET* genes, respectively. The *MdSWEET2* genes had nine homologs, *MdSWEET2a*, *MdSWEET2b*, *MdSWEET2c*, *MdSWEET2d*, *MdSWEET2e*, *MdSWEET2f*, *MdSWEET2g*, *MdSWEET2h*, and *MdSWEET2i*, while only one copy was observed for *MdSWEET1*, *MdSWEET8*, *MdSWEET11*, and *MdSWEET17*. The remaining *MdSWEET* genes, *MdSWEET2*, *MdSWEET5*, *MdSWEET7*, *MdSWEET9*, *MdSWEET10*, *MdSWEET12*, and *MdSWEET15* contained two homologs.

### Expression profiling of *MdSWEET* genes in fruit at different developmental stages

**Fig. 3:**
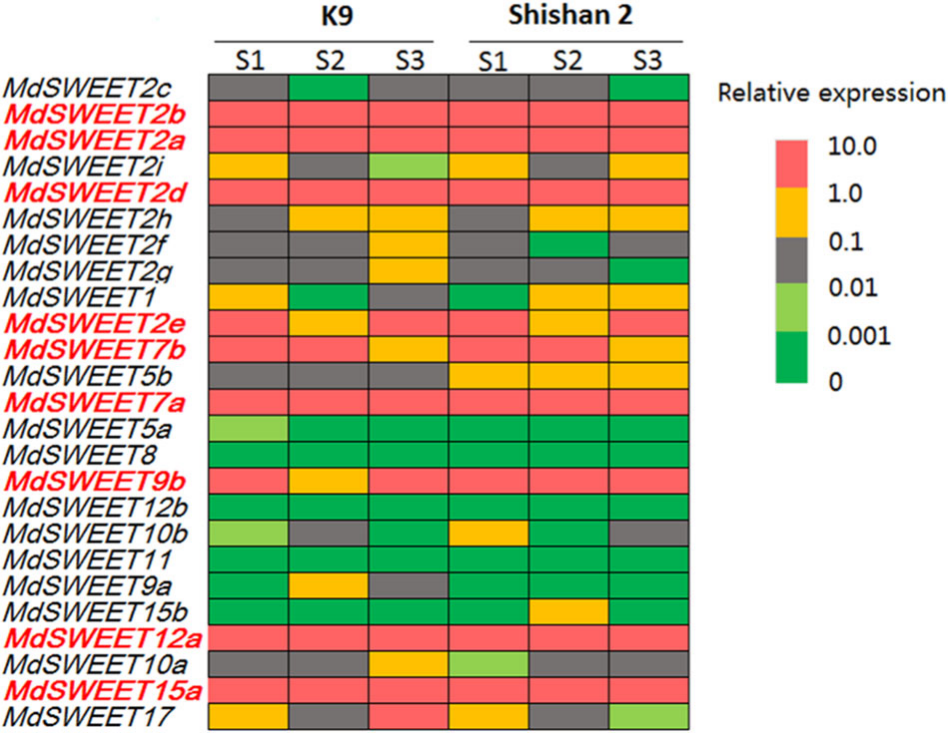
Expression profiles of *MdSWEETs* in fruits of two cultivars K9 and Shishan 2 at different developmental stages. The heat map was constructed based on relative expression levels of the *MdSWEETs*. S1, S2, and S3 represent young fruitlets, expanding fruits, and mature fruits, which were collected 30, 60, and 90 days after full bloom, respectively. The *MdSWEET* genes showing high expression throughout fruit development are highlighted in red color. The different colors represent different levels of gene expression, with the red color representing the highest values of gene expression, followed by yellow, gray, light green, and dark green

To identify *SWEET* genes that are potentially involved in fruit sugar accumulation, we investigated the expression profiling of the *MdSWEET* genes in fruits of two cultivars, K9 and Shishan 2, at different developmental stages, including juvenile, expanding, and mature stages (Fig. 3). Of the 25 *MdSWEET* genes, 16 showed extremely low expression throughout fruit development, with relative expression levels ranging from 0 to 0.1. In contrast, 9 *MdSWEET* genes, *MdSWEET2a*, *MdSWEET2b*, *MdSWEET2d*, *MdSWEET2e*, *MdSWEET7a*, *MdSWEET7b*, *MdSWEET9b*, *MdSWEET12a*, and *MdSWEET15a*, were highly expressed in fruits at all the three stages tested. Hence, these 9 *MdSWEET* genes were assumed to be potential candidates related to fruit sugar accumulation, and were further subjected to develop gene-tagged markers for evaluation of their genetic association with fruit sugar content.

### Development of gene-tagged markers for *SWEET*s with high expression in fruit and their polymorphisms in a collection of apple cultivars

Two types of molecular markers, SSR and CAPS, were developed for the nine *MdSWEET* genes mentioned above (Table 1). The SSR markers for two *SWEET* genes, *MdSWEET7a* and *MdSWEET9b*, were developed based on a (CT)_*n*_ microsatellite located in the second intron, whereas dinucleotide microsatellites, including (AG)_*n*_, (GA)_*n*_, (AT)_*n*_, (CT)_*n*_, and (TA)_*n*_, located upstream of the start codon were used to develop SSR markers for six *SWEET* genes, *MdSWEET2a*, *MdSWEET2b*, *MdSWEET2d*, *MdSWEET2e*, *MdSWEET7b*, and *MdSWEET12a* (Table 1). A T/C single-nucleotide polymorphism (SNP) in the first intron of *MdSWEET15a* was successfully used to develop a CAPS marker. PCR products harboring a “C” nucleotide at the polymorphic site could be digested with the *Nde*I enzyme, producing two fragments of 438 and 203 bp in size, while no digestion for PCR products harboring a “T” nucleotide.

**Table 1:** Primer sequences of molecular markers developed for nine *SWEET* genes highly expressed in fruit of apple

Gene	ID	LG	Gene tag			Primer sequence (5′–3′)		Expected size (bp)
			Type	Motif	Location	Forward	Reverse	
*MdSWEET2b*	MD11G1270800	11	SSR	(AG)_7_	93 bp of USC	TGAGGCAGAAACAATCATAAGGGTC	GAGCACGGAATTTGAAGCTGTAAAA	257
*MdSWEET2a*	MD03G1250600	3	SSR	(GA)_11_	102 bp of USC	ATACCGAGGAACTGTAGGACCAAGC	CTCCACACTAAACAACCAGAAAGCA	335
*MdSWEET2d*	MD05G1293100	5	SSR	(AT)_8_	664 bp of USC	CATTCAATTTATTCGACCGGACGAC	TGGGTTCATCCCTCACTTTCACTCA	270
*MdSWEET2e*	MD10G1269300	10	SSR	(AT)_7_	4203 bp of USC	GTGAGCCCACAACTAATCCCAT	CTTGTGCGTAGGAATCCCGATA	219
*MdSWEET7b*	MD06G1112000	6	SSR	(CT)_17_	192 bp of USC	GGGTTTTGAGAATCTTGAGGGTAGG	TTTGATGGGTTGGACTGTAACTTGC	251
*MdSWEET9b*	MD04G1236000	4	SSR	(CT)_19_	Second intron	GCGCCAATGTAAGACCCTTTACTTT	CTGACCTTGTCCTTCTTGGATGCGTA	348
*MdSWEET7a*	MD11G1299200	11	SSR	(CT)_14_	Second intron	TTCTATCTCCCCTTCCCAAACTTCC	GCTAAACAGTGCCACTGCATAAGGT	340
*MdSWEET12a*	MD14G1151300	14	SSR	(TA)_10_	1902 bp of USC	ATGACAGGGCAACTTCAGGGT	CGTAATAGTCCTTTGCCCTCC	253
*MdSWEET15a*	MD16G1125300	16	CAPS	T/C	First intron	ACCTACCAATCCTCCATCTGTC	CCATCACAATAACTCACCTGCTTC	641

*USC* upstream of start codon

**Fig. 4:**
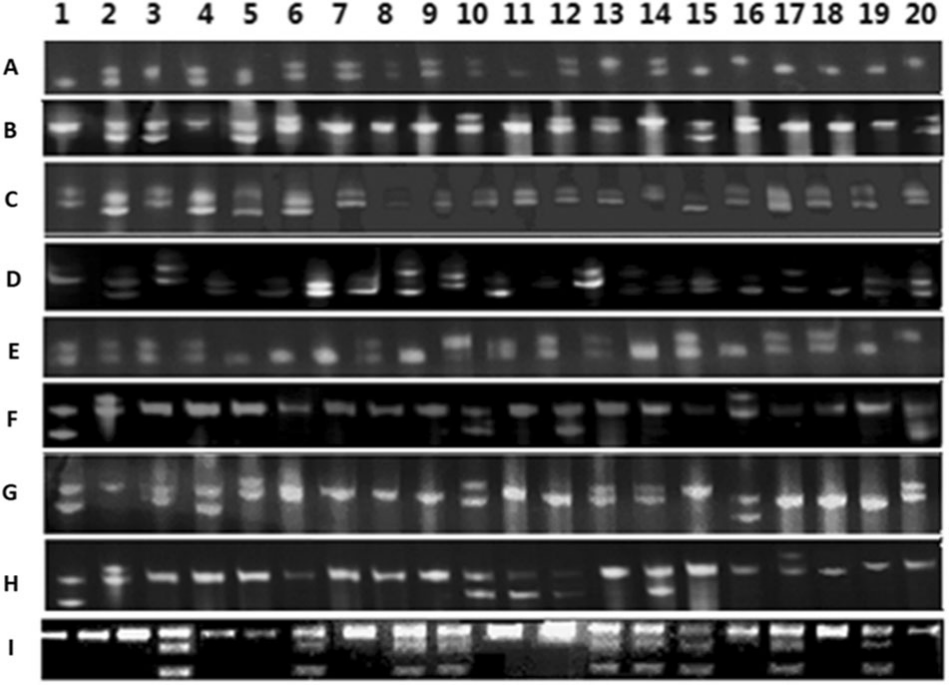
An example of genotyping of apple cultivars using molecular tags of the *MdSWEET* genes that were highly expressed during fruit development. **a**–**h** Polymorphic bands of SSR markers detected at the *MdSWEET2a*, *MdSWEET2b*, *MdSWEET2d*, *MdSWEET2e*, *MdSWEET7a*, *MdSWEET7b*, *MdSWEET9b*, and *MdSWEET12a* loci, respectively. **i** An agarose gel electrophoresis of PCR products derived from the first intron of *MdSWEET15a*, which are digested with the *Nde*I enzyme. Polymerase chain reactions products containing CATATG sequences can be digested, whereas PCR products with TATATG sequences cannot be digested. 1: Laidi; 2: Fulaibao; 3: Zaoshengchi; 4: Qingxiangjiao; 5: Beinaoni; 6: Fujin; 7: Qingguan; 8: Xingcheng 2310; 9: Bankeluofute; 10: Senmalan; 11: Yeweilin; 12: Kangbingjinguan 51; 13: Zhanxuan 14; 14: Xingcheng 1518; 15: Zhongxin; 16: Zhanxuan 4; 17: Hebeikangbingjinguan; 18: Jinguang; 19: Lisijin; 20: Tianhongyu

These nine gene-tagged markers were subsequently used to screen a collection of 188 cultivars (Fig. 4). As a result, two alleles at each polymorphic locus were detected for three *MdSWEET* genes, *MdSWEET2a*, *MdSWEET12a*, and *MdSWEET7a*, while three alleles at each polymorphic locus were observed for five *MdSWEET* genes, *MdSWEET2b*, *MdSWEET2d*, *MdSWEET2e*, *MdSWEET7b*, and *MdSWEET9b*. Six genotypes derived from the random combination of three alleles were identified at each of the four gene loci, including *MdSWEET2b*, *MdSWEET2d*, *MdSWEET2e*, and *MdSWEET7b*, while only four genotypes were detected at the *MdSWEET9b* locus (Table S4). Three genotypes at each polymorphic locus were identified for three *MdSWEET* genes, *MdSWEET2a*, *MdSWEET7a*, *and MdSWEET12a*. By contrast, the CAPS marker revealed only two genotypes, T/T and T/C, and the homologous genotype C/C was not identified in all the cultivars tested.

### Association between *MdSWEET* genes and fruit sugar accumulation in apple

**Fig. 5:**
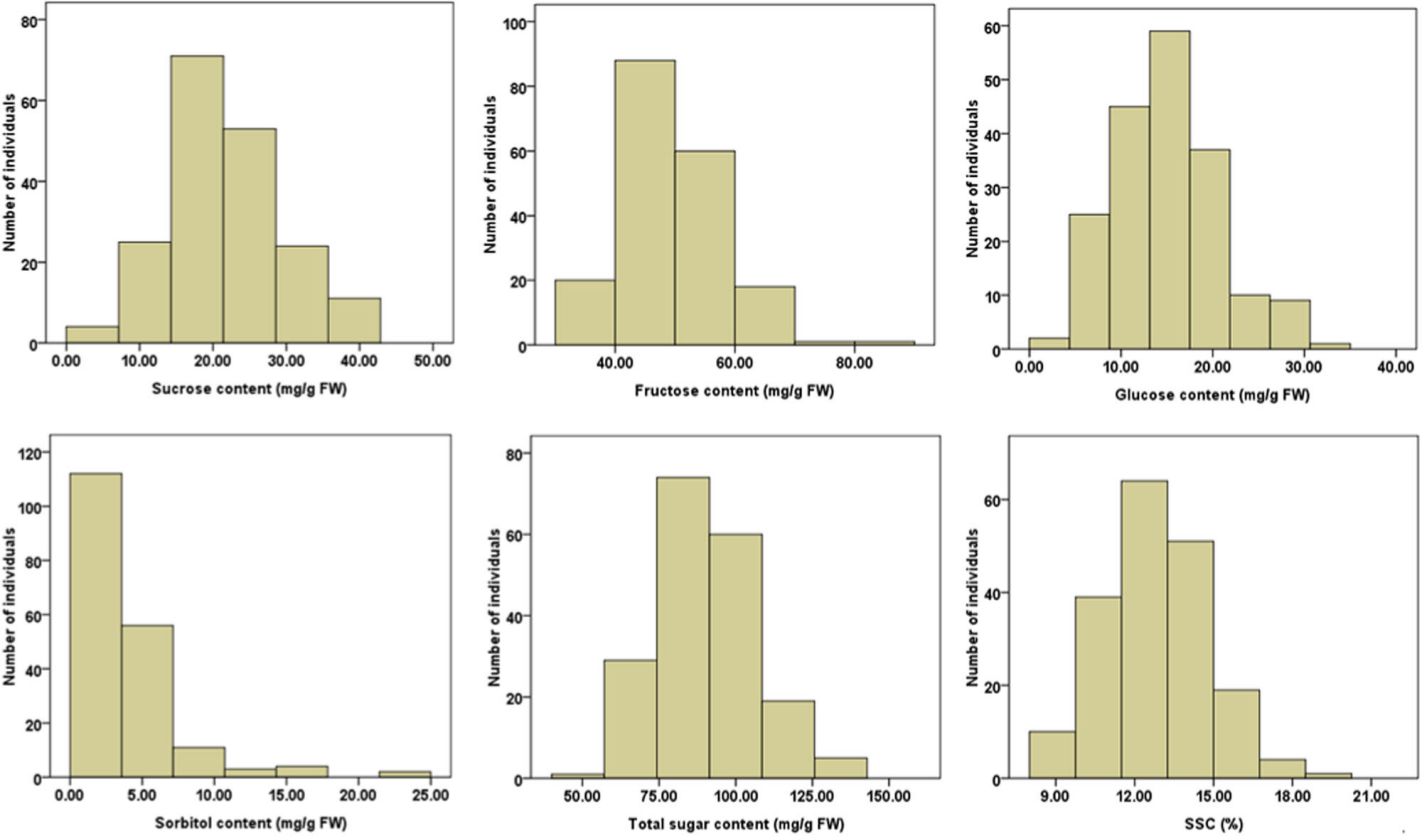
Distribution of fruit sugar parameters measured for mature fruits of apple germplasm .

SSC and soluble sugar content in mature fruit for all the tested cultivars are listed in Table S1 and their distributions are shown in Fig. 5. SSC and the contents of sucrose, fructose, glucose, and total sugar components showed a normal distribution, whereas the distribution of sorbitol content was skewed toward low sorbitol contents. A wide range was observed for the concentration of various sugar components, including sucrose (3.8–41.75 mg/g FW), glucose (3.86–31.80 mg/g FW), fructose (30.48–83.82 mg/g FW), sorbitol (0.14–22.62 mg/g FW), and total sugar components (47.93–142.40 mg/g FW), with an average of 21.65, 14.98, 49.07, 3.90, and 89.60 mg/g FW, respectively. SSC ranged from 7.96 to 20.02, with an average of 12.76. Overall, the apple cultivar collection has a great variation in fruit sugar content, which suggests that they are suitable for investigating genetic association of *MdSWEET* genes with fruit sugar accumulation.

**Fig. 6:**
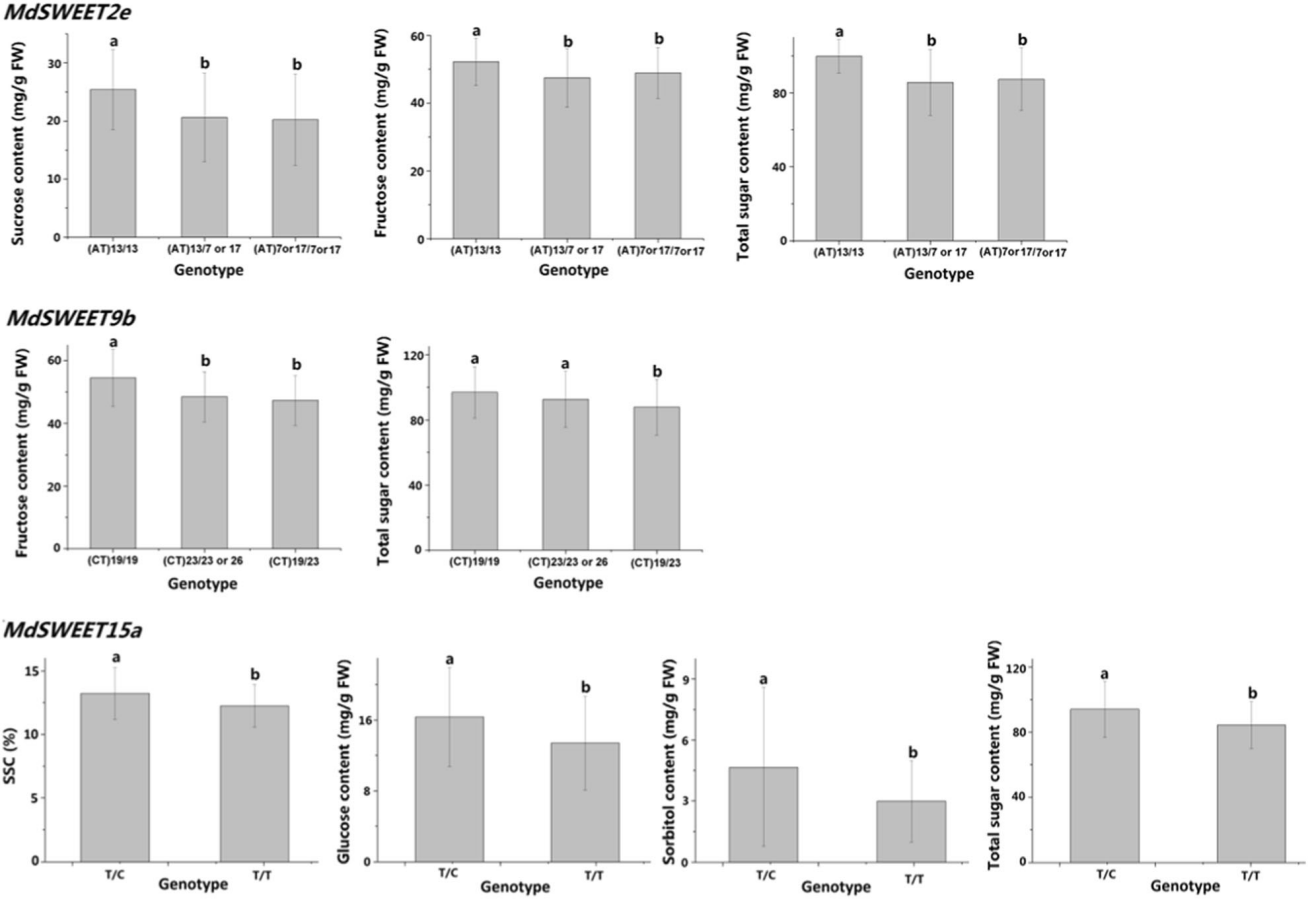
Mean values of soluble sugar contents in mature fruits of various genotypes at three different *MdSWEET* loci among the tested cultivars. Different lowercase letters indicate significant differences between genotypes (*t*-test, LSD test at *P* < 0.01)

Candidate gene-based association mapping was further performed to investigate association between the polymorphic loci of *MdSWEET* genes and fruit sugar accumulation in apple. As a result, three genes, *MdSWEET2e*, *MdSWEET9b*, and *MdSWEET15a*, showed a significant association with fruit sugar accumulation, whereas no significant association was observed for the remaining six genes, *MdSWEET2a*, *MdSWEET2b*, *MdSWEET2d*, *MdSWEET7a*, *MdSWEET7b*, and *MdSWEET12a* (Table 2). Based on the presence or absence of the (AT)_13_ allele on the *MdSWEET2e* locus, all the tested cultivars were grouped into three genotypes, (AT)_13/13_, (AT)_13/7 or 17_, and (AT)_7 or 17/7 or 17_. The (AT)_13/13_ genotype had significantly higher than both (AT)_13/7 or 17_ and (AT)_7 or 17/7 or 17_ genotypes for the sucrose, fructose, or total sugar content (Fig. 6). Similarly, all the cultivars were divided into three genotypes, (CT)_19/19_, (CT)_19/23_, and (CT)_23/23 or 26,_ based on the presence or absence of the (CT)_19_ allele on the *MdSWEET9b* locus. Cultivars with two (CT)_19_ alleles had significantly higher than cultivars with one or no (CT)_19_ allele for the fructose or total sugar content. Based on the polymorphic locus of *MdSWEET15a*, all the cultivars were assigned to two genotypes, T/T and T/C. The T/T genotype had significantly higher than the T/C genotype for SSC and the glucose, sorbitol, or total sugar content. However, the (AT)_13_ allele of *MdSWEET2e* and the (CT)_19_ allele of *MdSWEET9b* had no effect on the soluble solids, glucose, or sorbitol content and the soluble solids, sucrose, glucose, or sorbitol content, respectively (data not shown). Similarly, the polymorphic locus of *MdSWEET15a* had no effect on accumulation of sucrose and fructose.

**Table 2:** The probability value (*P*-value) of association between polymorphic loci of *MdSWEET* genes and soluble sugar content in mature fruits of 188 apple cultivars

Gene	Average SSC^a^	Average content of soluble sugar components (mg/g FW)^a^				
		Sucrose	Glucose	Fructose	Sorbitol	Total
*MdSWEET7b*	0.3461	0.4660	0.5707	0.9866	0.4530	0.9098
*MdSWEET2d*	0.0596	0.1501	0.7462	0.4083	0.8425	0.2916
*MdSWEET2b*	0.2998	0.3007	0.0256	0.8808	0.6281	0.9957
*MdSWEET2e*	0.0177	**0.0001**	0.0642	**0.0083**	0.0111	**0.0001**
*MdSWEET12a*	0.8595	0.8146	0.9991	0.4798	0.1514	0.8063
*MdSWEET9b*	0.0198	0.8811	0.1550	**0.0001**	0.4027	**0.0085**
*MdSWEET2a*	0.6065	0.9280	0.8084	0.0365	0.9427	0.6298
*MdSWEET7a*	0.0838	0.0966	0.9905	0.3783	0.0110	0.1418
*MdSWEET15a*	**0.0005**	0.0660	**0.0005**	0.0109	**0.0019**	**0.0001**

^a^The *P*-values < 0.01 are highlighted in bold

Of the above three genes associated with fruit sugar accumulation, *MdSWEET15a* had relatively higher contributions to the observed phenotypic variation, and accounted for 6.4%, 6.8%, 5.7%, and 8.4% of the observed phenotypic variation in the soluble solids, glucose, sorbitol and total sugar content, respectively. The *MdSWEET9b* gene accounted for 6.6% and 2.5% of the observed phenotypic variation in the fructose and total sugar content, respectively. By contrast, *MdSWEET2e* had lower contribution, accounting for 0.7%, 2.7%, and 3.6% of the observed phenotypic variation in the sucrose, fructose, and total sugar content, respectively.

Taken together, all the above results suggest that one clade II *SWEET* gene, *MdSWEET2e*, and two clade III *SWEET* genes, *MdSWEET9b* and *MdSWEET15a*, are genetically associated with sugar content in mature apple fruit, with *MdSWEET15a* and *MdSWEET9b* showing relatively higher contribution.

## DISCUSSION

Sugar transporters play a crucial role in plant growth and development as they mediate sugar uptake or release from cells or subcellular compartments^21^. *SWEET*s are newly identified sugar transporters and they are critical for sugar transportation and sink tissue development^17,22,42^. Although preliminary analyses of the *SWEET* gene family have been reported in several fruit crops^33–35^, little is known about the role of *SWEET*s in fruit sugar accumulation. In this study, we report for the first time genetic association of the *SWEET* genes with fruit sugar content in apple. Our study indicates that developing gene-tagged markers is an efficient way to investigate gene functionality, and the gene-tagged markers can be directly used in breeding programs if they are associated with horticultural traits of interest.

### Duplication of *SWEET*s in the apple genome

Gene duplication is a major driving force for recruitment of genes in plants. Apple is diploid, with an autopolyploidization origin^36,43^. Here our study reveals 25 *SWEET*s in the apple genome, which is inconsistent with a previous report that identifies a total of 29 apple *SWEET*s^33^. This inconsistency is likely due to the fact that our analysis is based on of the draft genome sequence of “Golden delicious” doubled-haploid tree (GDDH13 V1.1)^38^, while the genome sequence of apple cv. Golden Delicious^36^ was used in the study reported by Wei et al^33^. All the *MdSWEET* genes except *MdSWEET8* are located on homologous pairs of chromosomes. For example, chromosomes 6 and 14 are homologous pairs and both contain one copy of each of the four *SWEET* genes, *MdSWEET7*, *MdSWEET12*, *MdSWEET10*, and *MdSWEET5*. The *MdSWEET9* gene has two homologs, which are separately located on the bottom of two homologously paired chromosomes 4 and 12. Chromosomes 5 and 10 are homologous pairs and both contain four *MdSWEET* genes. These results unambiguously demonstrate that duplication of *SWEET* genes is related to whole-genome duplication (WGD).

Chromosome 3 is homologous to chromosome 11. Chromosome 3 contains a single *SWEET* gene *MdSWEET2a* on the bottom chromosome, whilst a cluster of two *SWEET2* genes, *MdSWEET2b* and *MdSWEET11*, are found on the bottom of chromosome 11. Similarly, chromosomes 13 and 16 are homologous pairs. A cluster of two *SWEET2* genes, *MdSWEET15b* and *MdSWEET17*, are found on the top of chromosome 13, whilst only single *SWEET* gene *MdSWEET15a* located on the homologous region of chromosome 16. These results also suggest that tandem duplication of *SWEET* genes has probably occurred on chromosomes 11 and 13. In addition, it is worth noting that one *SWEET* gene, *MdSWEET8*, is located on the bottom of chromosome 17, but no *SWEET* gene was found on chromosome 9 that is homologous to chromosome 17. Since duplicated gene copies following WGD are prone to be rapidly lost^44^, it is reasonable to speculate that a *SWEET* gene on chromosome 9 may have been lost in the ancestor of apple.

Most WGD-derived duplicated genes are prone to diverge in expression^45^. This case is also detected for the apple *SWEET* genes. For example, two *SWEET* homologs, *MdSWEET15a* and *MdSWEET15b*, which are located on homologous pair of chromosomes 16 and 13, respectively, have undergone divergence in expression. *MdSWEET15a* is highly expressed throughout fruit development, whilst *MdSWEET15b* shows no expression in fruit. Similarly, *MdSWEET9a* and *MdSWEET9b* are located on homologous pair of chromosomes 12 and 4, respectively. *MdSWEET9b* shows high expression throughout fruit development, but *MdSWEET9a* with no expression in fruit. In addition, expression divergence was also observed for tandem duplicated *SWEET* genes. For example, *MdSWEET2b* and *MdSWEET11* are clustered on the bottom of chromosome 11. *MdSWEET2b* is highly expressed throughout fruit development, but *MdSWEET11* shows no expression in fruit. However, it is unclear whether or not the remaining 16 *MdSWEET* genes with extremely low expression in fruit have also diverged in expression.

Taken together, all the results above suggest that the *SWEET* genes in apple have undergone polyploidization and/or segmental duplication during the process of speciation, and some duplicated *SWEET* genes have diverged in expression.

### Candidate *MdSWEET*s involved in the regulation of fruit sugar accumulation in apple

Measurement of soluble sugar content in mature fruits of 188 apple cultivars reveals that the average concentration of fructose is over twofold higher than those of sucrose, glucose, and sorbitol, which confirms our previous report of fructose being the major sugar component in mature apple fruit^37^. Genotyping of the apple cultivar collection using nine gene-tagged molecular markers further indicates that three *MdSWEET* genes, *MdSWEET2e*, *MdSWEET15a*, and *MdSWEET9b*, are associated with fruit sugar accumulation. Six genotypes produced by three alleles at single polymorphic locus were detected for four *SWEET* genes, *MdSWEET7b*, *MdSWEET2d*, *MdSWEET2b*, and *MdSWEET2e*, and three genotypes arisen from two alleles at single polymorphic locus were observed for three *SWEET* genes, *MdSWEET12a*, *MdSWEET2a*, and *MdSWEET7a*. In contrast, the (CT)_26/26_ and (CT)_19/26_ genotypes and the C/C genotype were not found at the *MdSWEET9b* or *MdSWEET15a* loci, respectively, in the apple cultivar collection. Since selection is well-known to be a directional process that results in changes in the frequency of various genotypes in the population, the *MdSWEET15a* and *MdSWEET9b* loci have probably undergone selection during the process of apple domestication.

Linkage mapping of quantitative trait loci (QTLs) for sugar content have revealed many QTLs with minor effects on all linkage groups (LGs) except LG7 and LG17^46–49^. *MdSWEET15a* is located on the region of LG16, which contains several QTLs for Brix and the amount of sorbitol and fructose^48,50^. Similarly, *MdSWEET9b* is located on the region of LG4 harboring a QTL for individual sugar content^49^. By contrast, *MdSWEET2e* is located far away (approximately 11.2 Mb) from the region of LG10 harboring a QTL for Brix and sucrose content. In addition, *MdSWEET2e* has a relative smaller contribution to phenotypic variation in sugar content compared with both *MdSWEET15a* and *MdSWEET9b*.

Taken together, all these results above suggest that *MdSWEET15a* and *MdSWEET9b* are likely candidates involved in sugar accumulation in apple fruit. In peach, two major QTLs for fruit sugar content have been reported on the top of LG4 and the bottom of LG5, respectively^51^. Interestingly, these two QTL regions both contain *SWEET* genes, based on the draft genome sequence of peach cv. Lovell^50^. The two QTL regions on peach LG4 and LG5 correspond to syntenic blocks on apple LG3 and LG6, respectively, which harbor QTLs for sugar content^52^. Thus, it seems that *SWEET* genes may also function as candidates for sugar accumulation in peach and other Rosaceae fruit crops.

Both *MdSWEET9b* and *MdSWEET15a* belong to the clade III *SWEET*s that are proved to be efficient SUTs^11^. In this study, the cultivars with the T/C genotype at the *MdSWEET15a* locus have higher average sucrose content in mature fruit compared with those with the T/T genotype. Similarly, the cultivars with two (CT)_19_ alleles have higher average sucrose content in mature fruit than those with one or no (CT)_19_ allele. Thus, the clade III *SWEET* genes in apple, similar to clade III *SWEET*s in *Arabidopsis*^9^, may be involved in sucrose transportation. However, the difference in average sucrose content between various genotypes in the *MdSWEET9b* or *MdSWEET15a* loci does not reach a significance level (*P* < 0.01). This might be partially due to the reason that sucrose in the vacuolar has a trend to conversion into hexoses, resulting in fructose being the major sugar component in mature apple fruit^37^.

Besides *MdSWEET15a* and *MdSWEET9b*, two additional *SWEET* genes, *MdSWEET2a* and *MdSWEET2b*, are also located on the regions of LGs 3 and 11, respectively, which contain QTLs for fruit sorbitol content in apple^53^. However, these two *MdSWEET* genes both have no significant association with sorbitol content. This could be attributed to certain process of sugar metabolism in fruit, where sorbitol unloaded from leaves into the fruit is converted to fructose or glucose^54^. It is worth noting that gene-tagged markers developed in this study may not correspond to functional variants that account for associations with sugar content. Thus, further studies are still needed to ascertain whether *MdSWEET2a* and *MdSWEET2b* have an influence on fruit sugar accumulation in apple.

Marker-assisted selection (MAS) is a valuable tool in breeding programs of plants, particularly fruit crops. In this study, a T/C SNP in the first intron of *MdSWEET15a* accounts for 6–8% of phenotypic variation for SSC (6.4%) and total sugar content (8.4%) among apple germplasm, while a (CT)_*n*_ SSR locus in the second intron of *MdSWEET9b* explains approximately 7% of phenotypic variation for the concentration of fructose, the major sugar component in apple fruit. Since these two gene-tagged makers account for considerable phenotypic variation, they can serve as efficient tools for genetic improvement of fruit sweetness in apple-breeding programs with MAS.

In summary, our study suggests that both *MdSWEET9b* and *MdSWEET15a* are likely candidates regulating fruit sugar accumulation in apple. It is worthy of study in the future to clarify the functions of *MdSWEET9b* and *MdSWEET15a*.

## Electronic supplementary material

Table S1 to Table S4(DOCX 76 kb)
