# Supplementary material for: Developing gene-tagged molecular markers for evaluation of genetic association of apple SWEET genes with fruit sugar accumulation
Source: Hortic Res. 2018 Mar 20;5:14. doi: 10.1038/s41438-018-0024-3 (PMC5859117; doi:10.1038/s41438-018-0024-3)
Supplement: Supplementary file 1 — Table S1 to Table S4(DOCX 76 kb) [file 41438_2018_24_MOESM1_ESM.docx]

Table S1 Soluble sugar characteristics of mature fruit for 188 apple cultivars

| Variety | SSC (%) | Soluble sugar component content (mg/g FW) | | | | |
| --- | --- | --- | --- | --- | --- | --- |
|  |  | Sucrose | Glucose | Fructose | Sorbitol | Total |
| 132 | 10.4 | 14.98 | 16.34 | 50.50 | 1.69 | 83.52 |
| 18688 | 13.3 | 28.59 | 17.34 | 44.23 | 2.85 | 93.01 |
| 2336 Qinguang | 10.8 | 17.36 | 15.18 | 47.28 | 1.35 | 81.17 |
| 4F18 | 12.6 | 19.34 | 16.24 | 46.62 | 2.46 | 84.66 |
| 60-15-30 | 13.1 | 15.54 | 12.62 | 48.61 | 4.02 | 80.79 |
| Ⅰ11－2 | 14.0 | 19.60 | 28.56 | 40.83 | 3.90 | 92.90 |
| Aihong | 9.1 | 18.87 | 16.98 | 43.79 | 1.59 | 81.24 |
| Aiwq | 14.9 | 19.54 | 12.18 | 47.77 | 4.53 | 84.02 |
| Alindun | 12.1 | 22.74 | 9.13 | 42.24 | 3.52 | 77.62 |
| Aozhou | 10.3 | 12.26 | 15.53 | 50.89 | 1.83 | 80.52 |
| Ayiwaniya | 13.4 | 32.01 | 9.01 | 58.28 | 6.51 | 105.81 |
| B Jinguan | 14.1 | 22.10 | 15.40 | 53.39 | 2.04 | 92.93 |
| Bankeluofute | 12.5 | 23.09 | 6.69 | 58.74 | 3.24 | 91.77 |
| Baoman | 13.2 | 36.24 | 9.07 | 52.66 | 5.05 | 103.02 |
| Baosikupu | 14.5 | 32.22 | 12.63 | 54.23 | 6.52 | 105.60 |
| Behene | 14.4 | 36.67 | 19.48 | 53.48 | 3.22 | 112.86 |
| Beidang | 14.7 | 41.75 | 8.50 | 51.41 | 6.22 | 107.89 |
| Beierpusi | 13.2 | 15.37 | 10.42 | 51.48 | 4.09 | 81.36 |
| Beinaoni | 13.5 | 16.72 | 8.99 | 35.28 | 0.48 | 61.48 |
| Bianqiangzi 1 | 11.8 | 23.45 | 19.73 | 43.22 | 2.59 | 88.99 |
| Bo 19 | 12.5 | 19.59 | 10.02 | 49.78 | 3.54 | 82.91 |
| Bo 8 | 11.5 | 20.26 | 9.08 | 47.22 | 4.40 | 80.97 |
| Chaersiluosi | 13.7 | 30.02 | 11.34 | 38.99 | 2.53 | 82.88 |
| Changhong | 13.1 | 21.34 | 11.73 | 49.31 | 2.65 | 85.04 |
| Chaohong | 12.5 | 28.81 | 15.11 | 51.98 | 1.57 | 97.47 |
| Chilong | 12.0 | 21.42 | 16.17 | 43.13 | 3.81 | 84.53 |
| Cuiyu | 15.2 | 22.02 | 22.83 | 41.95 | 2.55 | 89.35 |
| Dabinai | 13.6 | 21.63 | 15.62 | 45.78 | 2.74 | 85.78 |
| Daguoguang | 11.3 | 23.00 | 4.38 | 51.89 | 2.32 | 81.60 |
| Dajin | 13.6 | 12.80 | 16.27 | 44.38 | 3.14 | 76.58 |
| Danxia | 13.9 | 21.35 | 12.98 | 48.41 | 2.02 | 84.76 |
| De 14 | 10.0 | 19.32 | 12.97 | 42.90 | 1.91 | 77.10 |
| De 2 | 12.3 | 31.05 | 11.09 | 50.10 | 2.40 | 94.63 |
| Dinghong | 14.7 | 34.53 | 19.57 | 48.53 | 4.63 | 107.26 |
| Dongchengguan 13 | 11.6 | 33.57 | 20.71 | 49.19 | 7.12 | 110.60 |
| Duanzhiluao | 11.5 | 20.37 | 18.01 | 52.59 | 5.28 | 96.24 |
| Enweierjinaisheng | 12.7 | 26.26 | 14.18 | 51.06 | 1.62 | 93.11 |
| Fa 5 | 14.2 | 25.17 | 5.31 | 37.52 | 7.01 | 75.00 |
| Fengcunfushi | 12.3 | 17.62 | 13.20 | 46.72 | 1.95 | 79.49 |
| Fenghuangnuanhaitangguo | 11.6 | 16.26 | 13.00 | 50.56 | 4.52 | 84.34 |
| Fuhuapi | 11.7 | 26.41 | 8.99 | 52.28 | 14.67 | 102.35 |
| Fujin | 12.7 | 14.81 | 7.49 | 39.94 | 1.70 | 63.95 |
| Fulaibao | 11.0 | 12.80 | 8.40 | 37.13 | 1.06 | 59.39 |
| Fuqiu | 10.7 | 13.55 | 15.02 | 45.12 | 2.10 | 75.78 |
| Fushan 5 | 11.1 | 28.01 | 9.25 | 48.60 | 2.94 | 88.79 |
| Gaoqiu | 14.0 | 15.80 | 14.66 | 47.37 | 3.53 | 81.36 |
| Generos | 14.9 | 23.48 | 9.58 | 39.28 | 5.51 | 77.85 |
| Gudeboge | 15.0 | 26.80 | 27.50 | 61.11 | 8.79 | 124.20 |
| Guoqin | 14.3 | 30.03 | 18.99 | 46.59 | 7.53 | 103.13 |
| Hadibolaite | 9.6 | 10.28 | 16.92 | 43.68 | 0.82 | 71.71 |
| Hahong | 10.8 | 9.80 | 18.29 | 46.31 | 1.21 | 75.61 |
| Hebeikangbingjinguan | 15.8 | 34.39 | 13.69 | 64.35 | 5.72 | 118.15 |
| Helasang | 10.5 | 10.18 | 17.15 | 48.65 | 2.02 | 78.00 |
| Hesefengli | 10.3 | 12.93 | 7.94 | 42.71 | 0.82 | 64.39 |
| Holly（Hony） | 14.3 | 39.19 | 26.41 | 65.11 | 11.69 | 142.40 |
| Honglu | 11.8 | 27.05 | 12.09 | 46.11 | 1.10 | 86.34 |
| Hongxiezi | 12.7 | 23.99 | 7.93 | 44.71 | 2.44 | 79.07 |
| Hongxue | 11.3 | 19.63 | 15.88 | 58.10 | 1.10 | 94.71 |
| Hongyindu | 13.6 | 33.58 | 15.84 | 48.81 | 4.08 | 102.31 |
| Huangjin | 8.0 | 5.88 | 7.56 | 34.34 | 0.14 | 47.93 |
| Huangpi | 8.9 | 5.38 | 13.96 | 37.29 | 0.54 | 57.17 |
| Hui | 16.7 | 19.68 | 20.52 | 49.99 | 7.42 | 97.61 |
| Jiabukajinguang | 13.1 | 31.17 | 9.11 | 53.87 | 1.64 | 95.79 |
| Jie 15 | 8.7 | 11.27 | 16.79 | 37.58 | 0.86 | 66.49 |
| Jie 9 | 11.4 | 17.15 | 14.89 | 45.54 | 1.37 | 78.95 |
| Jieba | 12.9 | 25.22 | 9.18 | 55.78 | 3.18 | 93.36 |
| Jinaisheng | 10.4 | 16.03 | 11.98 | 65.36 | 1.01 | 94.39 |
| Jinguang | 11.3 | 13.20 | 14.70 | 35.65 | 1.15 | 64.69 |
| Jinguang | 14.5 | 35.69 | 21.57 | 56.62 | 5.43 | 119.31 |
| Jinguanyouxi | 12.5 | 30.67 | 15.05 | 54.37 | 1.51 | 101.61 |
| Jinyu | 12.6 | 24.43 | 18.71 | 47.75 | 5.03 | 95.91 |
| Jinyun | 13.2 | 37.74 | 15.83 | 45.45 | 1.52 | 100.53 |
| K12 | 13.2 | 25.09 | 9.86 | 53.72 | 2.14 | 90.81 |
| K9 | 14.5 | 18.84 | 14.93 | 41.16 | 7.39 | 82.33 |
| Kahong | 12.9 | 26.67 | 18.55 | 48.67 | 5.94 | 99.83 |
| Kangbingjinguan 51 | 11.5 | 24.91 | 15.51 | 50.22 | 1.74 | 92.38 |
| Kangtunduanzhi | 9.7 | 17.38 | 14.40 | 45.88 | 1.20 | 78.87 |
| Kelapu | 12.0 | 25.63 | 7.06 | 50.69 | 1.12 | 84.50 |
| Keluodeng | 12.7 | 20.72 | 16.61 | 36.78 | 1.01 | 75.11 |
| Kosttiq | 14.1 | 14.76 | 22.63 | 63.65 | 4.05 | 105.08 |
| Kuihua | 13.3 | 15.10 | 9.02 | 48.91 | 4.66 | 77.70 |
| Kuluona | 17.1 | 30.18 | 15.90 | 50.49 | 14.36 | 110.93 |
| Kunmasi | 11.1 | 18.25 | 7.24 | 42.20 | 2.42 | 70.12 |
| Labao | 10.2 | 17.00 | 7.70 | 47.00 | 1.67 | 73.36 |
| Laidi | 11.2 | 11.88 | 5.79 | 44.15 | 2.34 | 64.16 |
| Lanfengwang | 13.5 | 21.11 | 21.06 | 63.19 | 4.66 | 110.01 |
| Liberty | 13.9 | 16.38 | 19.69 | 52.60 | 4.08 | 92.75 |
| Lisijin | 15.1 | 38.86 | 15.68 | 49.94 | 15.83 | 120.31 |
| Liuyuyabian | 11.0 | 21.53 | 3.86 | 32.30 | 1.05 | 58.73 |
| Liuyuyabian | 14.0 | 26.14 | 19.17 | 51.37 | 4.96 | 101.65 |
| Lubi | 12.8 | 19.43 | 16.48 | 47.63 | 3.03 | 86.56 |
| Magu | 11.9 | 12.76 | 26.74 | 50.13 | 5.38 | 95.01 |
| Meiduan 1 | 9.3 | 11.99 | 12.69 | 40.57 | 1.81 | 67.05 |
| Meiguihong | 9.9 | 12.95 | 15.98 | 45.30 | 0.92 | 75.15 |
| Meina | 16.3 | 39.62 | 11.03 | 54.56 | 22.54 | 127.74 |
| Meixiang | 13.8 | 12.78 | 20.09 | 50.82 | 3.39 | 87.08 |
| Mesiketouming | 10.7 | 36.53 | 11.54 | 52.17 | 2.28 | 102.51 |
| Mianpinguo | 14.0 | 25.99 | 8.19 | 46.79 | 2.82 | 83.79 |
| Micui | 15.8 | 20.19 | 23.00 | 83.82 | 4.95 | 131.97 |
| Miguo | 14.1 | 21.44 | 12.15 | 42.57 | 4.74 | 80.91 |
| Mijin | 12.3 | 7.32 | 16.78 | 58.92 | 1.45 | 84.47 |
| Miqiulinjilian | 11.6 | 25.47 | 8.97 | 52.77 | 2.59 | 89.80 |
| N2 | 14.8 | 23.30 | 19.15 | 60.71 | 5.60 | 108.76 |
| Nanchengaijinguan | 16.1 | 24.46 | 21.91 | 52.33 | 5.74 | 104.44 |
| Nuosang | 10.5 | 19.15 | 17.73 | 42.30 | 2.03 | 81.22 |
| Onieffnin | 17.6 | 22.24 | 26.58 | 57.33 | 7.26 | 113.41 |
| Qiaonajin | 14.1 | 19.29 | 24.36 | 56.60 | 2.47 | 102.72 |
| Qiheduanjinguan | 14.5 | 20.85 | 21.28 | 64.14 | 2.90 | 109.17 |
| Qihu 1 | 10.5 | 9.74 | 17.91 | 45.22 | 1.64 | 74.52 |
| Qingdao 1 | 10.6 | 13.32 | 13.84 | 53.22 | 1.95 | 82.33 |
| Qingguan | 11.0 | 16.94 | 5.60 | 41.22 | 0.40 | 64.15 |
| Qingming | 12.6 | 25.23 | 18.02 | 57.46 | 3.00 | 103.71 |
| Qinguan | 10.8 | 14.35 | 16.03 | 42.46 | 2.39 | 75.23 |
| Qingxiangjiao | 10.7 | 14.91 | 9.07 | 35.86 | 0.55 | 60.40 |
| Qiujin | 15.3 | 19.00 | 11.31 | 41.05 | 8.48 | 79.83 |
| Qiujinxing | 15.2 | 27.17 | 6.95 | 60.97 | 4.88 | 99.98 |
| Rizhiwan | 12.0 | 16.80 | 15.94 | 37.57 | 1.14 | 71.45 |
| Ruidan | 13.8 | 30.11 | 12.81 | 43.13 | 4.29 | 90.34 |
| Ruiguang | 13.5 | 24.62 | 15.08 | 47.40 | 2.07 | 89.16 |
| Ruilianna | 16.0 | 26.54 | 17.53 | 59.07 | 5.33 | 108.46 |
| Senmalan | 11.5 | 16.84 | 11.02 | 62.51 | 1.65 | 92.03 |
| Shalatuoni | 15.9 | 40.54 | 29.61 | 55.23 | 8.87 | 134.25 |
| Shenglihongguan | 10.8 | 20.21 | 12.64 | 44.71 | 0.88 | 78.44 |
| Shiai | 14.6 | 32.47 | 15.81 | 42.37 | 5.82 | 96.47 |
| Shikou 26 | 13.9 | 25.85 | 15.82 | 48.55 | 5.76 | 95.98 |
| Sitakeaijinguan | 12.0 | 23.24 | 23.44 | 50.64 | 3.06 | 100.37 |
| Situonuowei | 11.1 | 20.05 | 5.27 | 40.09 | 1.56 | 66.97 |
| Suyisiliebo | 12.3 | 22.80 | 7.05 | 49.80 | 0.69 | 80.34 |
| Szampion(Szamlion | 13.3 | 24.52 | 15.34 | 51.31 | 4.12 | 95.30 |
| Taipingyangmeigui | 13.5 | 19.71 | 7.49 | 44.77 | 1.36 | 73.32 |
| Tianhongyu | 14.2 | 34.70 | 17.16 | 59.52 | 9.83 | 121.20 |
| Weiqinni | 17.6 | 25.54 | 31.80 | 60.17 | 15.59 | 133.10 |
| Weisitabeila | 12.4 | 15.18 | 12.91 | 48.14 | 1.56 | 77.80 |
| Weixishengming | 11.4 | 19.92 | 16.28 | 48.75 | 0.48 | 85.42 |
| Wuyue | 11.5 | 13.40 | 13.33 | 52.70 | 6.88 | 86.30 |
| Ximengfei | 11.8 | 21.04 | 17.12 | 45.64 | 2.01 | 85.81 |
| Xingcheng 1018 | 9.3 | 10.23 | 18.25 | 36.02 | 0.41 | 64.90 |
| Xingcheng 118 | 11.8 | 19.82 | 18.81 | 51.90 | 2.14 | 92.67 |
| Xingcheng 1211 | 11.2 | 18.58 | 13.87 | 44.86 | 3.16 | 80.46 |
| Xingcheng 1211 | 12.3 | 15.64 | 12.56 | 42.77 | 0.70 | 71.67 |
| Xingcheng 1518 | 12.9 | 38.11 | 19.99 | 51.57 | 7.71 | 117.38 |
| Xingcheng 159 | 13.8 | 15.96 | 11.01 | 34.76 | 4.25 | 65.98 |
| Xingcheng 174 | 11.8 | 12.22 | 21.97 | 49.90 | 6.72 | 90.80 |
| Xingcheng 2116 | 11.3 | 9.29 | 12.13 | 57.97 | 1.22 | 80.61 |
| Xingcheng 2117 | 15.2 | 21.94 | 8.61 | 64.76 | 6.33 | 101.64 |
| Xingcheng 220 | 13.0 | 23.01 | 19.65 | 55.17 | 9.03 | 106.86 |
| Xingcheng 231 | 12.6 | 19.69 | 15.59 | 63.19 | 1.57 | 100.04 |
| Xingcheng 250 | 17.2 | 8.17 | 29.65 | 63.39 | 12.74 | 113.95 |
| Xingcheng 2510 | 16.0 | 14.98 | 17.92 | 71.42 | 6.47 | 110.79 |
| Xingcheng 2614 | 13.9 | 12.71 | 17.46 | 48.19 | 1.43 | 79.80 |
| Xingcheng 300 | 15.1 | 24.82 | 27.86 | 41.53 | 7.13 | 101.34 |
| Xingcheng 319 | 14.3 | 14.63 | 12.23 | 51.92 | 1.50 | 80.28 |
| Xingcheng 423 | 14.0 | 32.79 | 18.59 | 60.43 | 5.16 | 116.97 |
| Xingcheng 52 | 11.8 | 3.80 | 19.69 | 44.29 | 0.85 | 68.62 |
| Xingcheng 77 | 10.7 | 14.39 | 20.40 | 41.67 | 1.94 | 78.40 |
| Xingcheng 79 | 14.0 | 17.57 | 13.29 | 57.42 | 5.12 | 93.40 |
| Xingcheng 923 | 12.7 | 14.98 | 23.75 | 53.75 | 2.87 | 95.35 |
| Xingcheng 93 | 9.6 | 4.85 | 20.32 | 46.36 | 0.82 | 72.36 |
| Xingcheng192 | 13.0 | 17.28 | 20.38 | 33.83 | 2.71 | 74.20 |
| Xingcheng2310 | 14.0 | 25.06 | 14.66 | 49.23 | 2.29 | 91.24 |
| Xingping | 15.0 | 26.10 | 19.05 | 47.43 | 5.70 | 98.28 |
| Xingxheng 1121 | 11.6 | 21.48 | 8.22 | 42.03 | 2.48 | 74.21 |
| Xinhong | 10.4 | 15.56 | 16.21 | 40.24 | 2.63 | 74.65 |
| Xinhongxing | 9.7 | 14.83 | 14.75 | 37.42 | 1.60 | 68.59 |
| Xinhongyu | 13.7 | 30.62 | 16.99 | 43.06 | 3.32 | 93.99 |
| Xinjiang327 | 10.1 | 14.84 | 15.80 | 44.40 | 1.18 | 76.21 |
| Xinjiangapple | 11.8 | 27.00 | 8.85 | 48.74 | 5.62 | 90.21 |
| Xinqiaonajin | 12.5 | 21.38 | 13.77 | 52.67 | 1.07 | 88.88 |
| Xinwojin | 10.0 | 18.34 | 9.93 | 44.57 | 2.10 | 74.95 |
| Xiteshisheng | 12.4 | 31.58 | 14.77 | 52.39 | 5.67 | 104.40 |
| Yanfu 1 | 15.6 | 27.83 | 23.47 | 51.41 | 5.35 | 108.07 |
| Yeweilin | 10.8 | 18.52 | 8.33 | 62.42 | 2.92 | 92.19 |
| Yidoujin | 14.1 | 28.82 | 21.51 | 41.69 | 4.13 | 96.15 |
| Yinggelan | 11.9 | 17.22 | 7.21 | 40.42 | 1.16 | 66.01 |
| Yingjin | 12.8 | 24.75 | 8.98 | 59.41 | 2.06 | 95.19 |
| Youjin | 11.9 | 26.47 | 17.74 | 51.89 | 3.28 | 99.37 |
| Youlixiang | 13.9 | 38.22 | 9.81 | 64.52 | 4.03 | 116.58 |
| Zakeliebieer | 12.6 | 13.47 | 17.40 | 53.53 | 11.76 | 96.16 |
| Zakeliebieer | 11.7 | 16.02 | 9.61 | 42.79 | 4.56 | 72.99 |
| Zaoshengchi | 10.8 | 23.25 | 4.29 | 30.48 | 1.70 | 59.73 |
| Zaoshenghezhinuan | 11.3 | 20.23 | 6.41 | 53.94 | 0.94 | 81.51 |
| Zhanxuan 14 | 15.0 | 25.31 | 18.27 | 41.63 | 7.31 | 92.52 |
| Zhanxuan 14 | 13.6 | 26.18 | 17.66 | 44.99 | 4.60 | 93.44 |
| Zhanxuan 4 | 20.0 | 30.31 | 15.73 | 49.24 | 22.62 | 117.90 |
| Zhongxin | 13.1 | 22.91 | 27.75 | 63.62 | 3.13 | 117.40 |
| Zhumaliya | 13.1 | 34.61 | 5.95 | 39.97 | 3.80 | 84.33 |
| Zhuoai 1 | 14.0 | 24.23 | 17.98 | 57.20 | 4.11 | 103.52 |
| Ziyu | 15.5 | 18.49 | 24.62 | 41.77 | 5.76 | 90.64 |

Table S2 Primer sequences of *MdSWEET* genes used for qRT-PCR analysis

| *SWEET* gene | Primer (5’ → 3’) | |
| --- | --- | --- |
|  | Forward | Reverse |
| *MdSWEET2a* | TGCACATGGTATGGCCTGC | ACCTTCCTCATGAGCACCTCT |
| *MdSWEET9b* | TGGCATCGCCCAAATGATCC | TTGTTCACACTGCCCGTCAA |
| *MdSWEET2i* | TCCAAATGGGTTTGGGAGTGG | ACGCCCATCTCCATTGATTCG |
| *MdSWEET2d* | ACTGCAGCCGGCAGGAATA | CAGGCATGCCATACCAGGAG |
| *MdSWEET2h* | AGTTTCTGCGATGGAGCCG | TCACTATAGGCATTCCATACCAGC |
| *MdSWEET2f* | TGCACATGGTATGGCCTGC | TTAAGCTCACAAGGACGACGG |
| *MdSWEET7b* | CCGTTGTTCTGAGAGGGAGC | CTATTCCGAATGGGCTGGGC |
| *MdSWEET12b* | TCTACGTTGCAACCCCAAACA | CATGGGAGCTTACAGCTGCTT |
| *MdSWEET10b* | TGAACGGTTTTAGTTACGCAGTG | GACACCAAGAGGTGCGACA |
| *MdSWEET5b* | TAGGCTCACAACGCCTAACG | CGCATGACTGTCAAGGGTGA |
| *MdSWEET2g* | CCGTTGTTCTGAGAGGGAGC | ACAACGGTTCTCTGGAGTTTCC |
| *MdSWEET1* | GTTGTTGTGTTCGCGTTCGT | CTTGACCAGTTTAGCGGCGA |
| *MdSWEET2e* | TATCAAACTTTCACTGATGCCGC | ATGCCACTCTTCACTATAGGCATT |
| *MdSWEET2c* | TCAATCGGAGCTGTTTTCCAGT | ACAGTTCCCTCTCATGGGCT |
| *MdSWEET2b* | TTCCACCTTCCTAATGAGCACC | ACAACGGTTCTCTGGAGTCCT |
| *MdSWEET11* | AATTCCATCGCCTGCGTCAT | TAGGTACGCCAAATCGGATCG |
| *MdSWEET9a* | CTACATTGCAGAGATGAAGAAGTTG | GTGTTCATCGTTTGCTTCGTAGA |
| *MdSWEET15b* | GTGCGCTCCGTACAGGAAT | TCAGGGTGAACAAACGGCAT |
| *MdSWEET17* | CTGCTCAACTCCTCCCTGTG | TACCCTCATTTTTGCCGGGG |
| *MdSWEET7a* | TTCCGAATGGGCTGGGCTTA | TTCTGATGAGTGCTGCCAGTC |
| *MdSWEET12a* | GCCTTTGGCATTCTAGGCAAC | CAGGATCACATGAGACTTGAGGA |
| *MdSWEET10a* | ATCAACTCGGTTGGCTGCG | CCATCATGCCGTAACTGAAAAGG |
| *MdSWEET5a* | TTGTCACCGAACTCGCCTAC | TCCCAACTACCAGAGACCGC |
| *MdSWEET15a* | CTAGTGGGGAGTGCATACCG | AACCAGATAACGGCGCTCAA |
| *MdSWEET8* | GCATTATCGGTACGTGCGCT | TCAGGGTGAACAAACGGCAT |

Table S3 Twenty-five *SWEET* genes in the apple genome and their structure feature of genomic DNA and amino acid sequences

| SWEET gene | GDR accession no. | Chr. | Transcript start | Transcript stop | Transcription direction | Length of gene | No. of exons | Size of ORF* (bp) | Protein size (aa) | No. of TMs* |
| --- | --- | --- | --- | --- | --- | --- | --- | --- | --- | --- |
| *MdSWEET2a* | MD03G1250600 | 3 | 33875915 | 33879929 | +strand | 4014 | 6 | 705 | 235 | 7 |
| *MdSWEET9b* | MD04G1236000 | 4 | 31488876 | 31490819 | -strand | 1944 | 6 | 801 | 267 | 7 |
| *MdSWEET2i* | MD05G1012200 | 5 | 2448222 | 2451303 | -strand | 3135 | 6 | 789 | 263 | 7 |
| *MdSWEET2d* | MD05G1293100 | 5 | 42452848 | 42454738 | -strand | 1858 | 6 | 699 | 233 | 7 |
| *MdSWEET2h* | MD05G1293200 | 5 | 42458344 | 42460525 | -strand | 2181 | 6 | 717 | 239 | 7 |
| *MdSWEET2f* | MD05G1293300 | 5 | 42489185 | 42491697 | -strand | 2513 | 6 | 696 | 232 | 7 |
| *MdSWEET7b* | MD06G1112000 | 6 | 25053642 | 25056504 | -strand | 2862 | 5 | 783 | 261 | 7 |
| *MdSWEET12b* | MD06G1136500 | 6 | 28124821 | 28127305 | +strand | 2484 | 6 | 894 | 298 | 7 |
| *MdSWEET10b* | MD06G1136600 | 6 | 28162839 | 28164846 | +strand | 2007 | 6 | 885 | 295 | 7 |
| *MdSWEET5b* | MD06G1176800 | 6 | 31681969 | 31683911 | -strand | 1942 | 5 | 645 | 215 | 6 |
| *MdSWEET2g* | MD10G1013100 | 10 | 1754882 | 1757564 | -strand | 2644 | 6 | 744 | 248 | 7 |
| *MdSWEET1* | MD10G1269100 | 10 | 36171364 | 36172894 | -strand | 1530 | 6 | 687 | 229 | 7 |
| *MdSWEET2e* | MD10G1269300 | 10 | 36182206 | 36184461 | -strand | 2255 | 6 | 714 | 238 | 7 |
| *MdSWEET2c* | MD10G1269400 | 10 | 36194260 | 36196760 | -strand | 2500 | 6 | 696 | 232 | 7 |
| *MdSWEET2b* | MD11G1270800 | 11 | 38631805 | 38635563 | +strand | 3759 | 6 | 705 | 235 | 7 |
| *MdSWEET11* | MD11G1299200 | 11 | 41575287 | 41576791 | -strand | 1505 | 5 | 1020 | 340 | 8 |
| *MdSWEET9a* | MD12G1255000 | 12 | 32322191 | 32323883 | -strand | 1693 | 6 | 702 | 234 | 6 |
| *MdSWEET15b* | MD13G1124300 | 13 | 9229495 | 9232078 | -strand | 2584 | 6 | 915 | 305 | 7 |
| *MdSWEET17* | MD13G1166800 | 13 | 13378189 | 13384879 | +strand | 6691 | 6 | 843 | 281 | 7 |
| *MdSWEET7a* | MD14G1133400 | 14 | 21124942 | 21127953 | -strand | 3012 | 5 | 783 | 261 | 7 |
| *MdSWEET12a* | MD14G1151300 | 14 | 24454255 | 24456823 | +strand | 2557 | 6 | 894 | 298 | 7 |
| *MdSWEET10a* | MD14G1151400 | 14 | 24479285 | 24481341 | +strand | 2056 | 6 | 885 | 295 | 7 |
| *MdSWEET5a* | MD14G1183000 | 14 | 27571175 | 27573168 | -strand | 1993 | 6 | 708 | 236 | 6 |
| *MdSWEET15a* | MD16G1125300 | 16 | 9081893 | 9084706 | -strand | 2814 | 6 | 912 | 304 | 7 |
| *MdSWEET8* | MD17G1035200 | 17 | 2521598 | 2523402 | -strand | 1805 | 6 | 750 | 250 | 7 |

*ORF: open reading frame; TMs: transmembrane domains.

Table S4 Genotypes at different *MdSWEET* loci for 188 apple accessions

|  | Genotypes | | | | | | | | |
| --- | --- | --- | --- | --- | --- | --- | --- | --- | --- |
| Cultivars | *MdSWEET7b* | *MdSWEET2d* | *MdSWEET2b* | *MdSWEET2e* | *MdSWEET12a* | *MdSWEET9b* | *MdSWEET2a* | *MdSWEET7a* | *MdSWEET15a* |
| Lisijin | (CT)_17/23_ | (AT)_13/16_ | (AG)_12/12_ | (AT)_7/13_ | (TA)_16/16_ | (CT)_19/23_ | (GA)_11/11_ | (CT)_14/20_ | T/C |
| Dongchengguan 13 | (CT)_23/23_ | (AT)_13/16_ | (AG)_12/12_ | (AT)_13/13_ | (TA)_16/16_ | (CT)_19/23_ | (GA)_16/16_ | (CT)_20/20_ | T/C |
| Shenglihongguan | (CT)_17/23_ | (AT)_13/16_ | (AG)_12/12_ | (AT)_7/13_ | (TA)_10/16_ | (CT)_19/23_ | (GA)_11/16_ | (CT)_14/14_ | T/C |
| Kahong | (CT)_17/23_ | (AT)_8/13_ | (AG)_12/12_ | (AT)_13/13_ | (TA)_16/16_ | (CT)_19/23_ | (GA)_11/16_ | (CT)_20/20_ | T/T |
| Xingcheng 1018 | (CT)_17/23_ | (AT)_13/16_ | (AG)_12/12_ | (AT)_7/17_ | (TA)_16/16_ | (CT)_19/23_ | (GA)_11/16_ | (CT)_14/20_ | T/T |
| Baoman | (CT)_17/17_ | (AT)_13/16_ | (AG)_7/12_ | (AT)_13/13_ | (TA)_16/16_ | (CT)_19/23_ | (GA)_11/16_ | (CT)_20/20_ | T/T |
| Chaersiluosi | (CT)_17/17_ | (AT)_13/16_ | (AG)_12/12_ | (AT)_7/13_ | (TA)_16/16_ | (CT)_23/26_ | (GA)_11/16_ | (CT)_20/20_ | T/T |
| Youjin | (CT)_29/29_ | (AT)_13/16_ | (AG)_12/12_ | (AT)_13/13_ | (TA)_10/16_ | (CT)_19/23_ | (GA)_16/16_ | (CT)_20/20_ | T/C |
| Shikou 26 | (CT)_17/23_ | (AT)_13/16_ | (AG)_12/12_ | (AT)_13/13_ | (TA)_10/16_ | (CT)_19/23_ | (GA)_11/16_ | (CT)_20/20_ | T/C |
| Dinghong | (CT)_23/23_ | (AT)_8/13_ | (AG)_7/12_ | (AT)_7/13_ | (TA)_10/16_ | (CT)_23/26_ | (GA)_11/16_ | (CT)_20/20_ | T/C |
| Labao | (CT)_17/23_ | (AT)_13/16_ | (AG)_12/12_ | (AT)_7/13_ | (TA)_10/16_ | (CT)_19/23_ | (GA)_11/16_ | (CT)_20/20_ | T/T |
| Alindun | (CT)_23/29_ | (AT)_16/16_ | (AG)_12/12_ | (AT)_17/17_ | (TA)_10/16_ | (CT)_23/26_ | (GA)_11/16_ | (CT)_14/14_ | T/T |
| Bo 8 | (CT)_17/23_ | (AT)_16/16_ | (AG)_7/12_ | (AT)_7/13_ | (TA)_10/16_ | (CT)_19/23_ | (GA)_11/16_ | (CT)_14/20_ | T/T |
| Jinguang | (CT)_23/29_ | (AT)_8/16_ | (AG)_12/12_ | (AT)_7/7_ | (TA)_10/10_ | (CT)_23/26_ | (GA)_16/16_ | (CT)_14/14_ | T/T |
| Fujin | (CT)_23/23_ | (AT)_13/16_ | (AG)_12/12_ | (AT)_7/7_ | (TA)_10/10_ | (CT)_23/26_ | (GA)_16/16_ | (CT)_14/20_ | T/T |
| Xingxheng 1121 | (CT)_23/23_ | (AT)_8/13_ | (AG)_12/12_ | (AT)_7/13_ | (TA)_10/16_ | (CT)_23/26_ | (GA)_16/16_ | (CT)_14/20_ | T/T |
| Bo 19 | (CT)_17/23_ | (AT)_8/13_ | (AG)_12/12_ | (AT)_13/17_ | (TA)_16/16_ | (CT)_19/23_ | (GA)_16/16_ | (CT)_20/20_ | T/C |
| Jinyun | (CT)_23/29_ | (AT)_16/16_ | (AG)_12/12_ | (AT)_13/13_ | (TA)_10/16_ | (CT)_19/23_ | (GA)_16/16_ | (CT)_20/20_ | T/C |
| Xingcheng 118 | (CT)_17/17_ | (AT)_13/16_ | (AG)_7/12_ | (AT)_13/13_ | (TA)_10/16_ | (CT)_19/19_ | (GA)_11/16_ | (CT)_20/20_ | T/T |
| Wuyue | (CT)_23/29_ | (AT)_13/16_ | (AG)_17/17_ | (AT)_7/13_ | (TA)_16/16_ | (CT)_19/23_ | (GA)_16/16_ | (CT)_20/20_ | T/T |
| Xingcheng 1211 | (CT)_23/23_ | (AT)_8/13_ | (AG)_12/12_ | (AT)_17/17_ | (TA)_10/16_ | (CT)_19/23_ | (GA)_11/16_ | (CT)_20/20_ | T/C |
| Jinyu | (CT)_17/23_ | (AT)_13/16_ | (AG)_12/12_ | (AT)_13/13_ | (TA)_10/16_ | (CT)_19/23_ | (GA)_11/16_ | (CT)_20/20_ | T/C |
| Honglu | (CT)_23/23_ | (AT)_16/16_ | (AG)_12/12_ | (AT)_7/7_ | (TA)_16/16_ | (CT)_19/23_ | (GA)_16/16_ | (CT)_14/20_ | T/T |
| Helasang | (CT)_23/29_ | (AT)_13/16_ | (AG)_12/12_ | (AT)_7/13_ | (TA)_10/16_ | (CT)_19/23_ | (GA)_16/16_ | (CT)_14/20_ | T/C |
| Xinjiangapple | (CT)_23/23_ | (AT)_8/13_ | (AG)_12/12_ | (AT)_13/13_ | (TA)_10/10_ | (CT)_19/23_ | (GA)_16/16_ | (CT)_20/20_ | T/T |
| Jie 15 | (CT)_23/29_ | (AT)_8/8_ | (AG)_7/12_ | (AT)_17/17_ | (TA)_16/16_ | (CT)_19/23_ | (GA)_11/16_ | (CT)_14/20_ | T/T |
| Mianpinguo | (CT)_17/23_ | (AT)_8/13_ | (AG)_12/12_ | (AT)_7/13_ | (TA)_16/16_ | (CT)_19/23_ | (GA)_11/16_ | (CT)_20/20_ | T/C |
| Beinaoni | (CT)_23/23_ | (AT)_8/13_ | (AG)_7/7_ | (AT)_7/13_ | (TA)_16/16_ | (CT)_19/23_ | (GA)_16/16_ | (CT)_14/20_ | T/T |
| Fa 5 | (CT)_23/23_ | (AT)_13/16_ | (AG)_12/12_ | (AT)_7/13_ | (TA)_10/16_ | (CT)_19/23_ | (GA)_11/16_ | (CT)_14/20_ | T/T |
| Kuluona | (CT)_23/29_ | (AT)_8/13_ | (AG)_12/12_ | (AT)_13/13_ | (TA)_16/16_ | (CT)_23/23_ | (GA)_16/16_ | (CT)_20/20_ | T/C |
| Jinguanyouxi | (CT)_29/29_ | (AT)_8/13_ | (AG)_12/12_ | (AT)_13/17_ | (TA)_10/16_ | (CT)_19/23_ | (GA)_16/16_ | (CT)_20/20_ | T/C |
| Laidi | (CT)_23/29_ | (AT)_13/13_ | (AG)_12/12_ | (AT)_7/13_ | (TA)_16/16_ | (CT)_19/23_ | (GA)_11/16_ | (CT)_20/20_ | T/T |
| N2 | (CT)_17/23_ | (AT)_8/13_ | (AG)_7/12_ | (AT)_7/13_ | (TA)_10/16_ | (CT)_23/23_ | (GA)_16/16_ | (CT)_20/20_ | T/T |
| Nuosang | (CT)_23/29_ | (AT)_8/13_ | (AG)_7/12_ | (AT)_7/13_ | (TA)_10/16_ | (CT)_19/23_ | (GA)_11/11_ | (CT)_14/14_ | T/T |
| Hebeikangbingjinguan | (CT)_23/29_ | (AT)_8/13_ | (AG)_12/17_ | (AT)_7/13_ | (TA)_16/16_ | (CT)_19/23_ | (GA)_16/16_ | (CT)_14/20_ | T/T |
| Zhanxuan 14 | (CT)_23/29_ | (AT)_13/16_ | (AG)_12/12_ | (AT)_13/13_ | (TA)_16/16_ | (CT)_19/23_ | (GA)_11/16_ | (CT)_20/20_ | T/C |
| Fulaibao | (CT)_23/29_ | (AT)_13/16_ | (AG)_12/12_ | (AT)_17/17_ | (TA)_16/16_ | (CT)_19/23_ | (GA)_16/16_ | (CT)_14/20_ | T/T |
| Kangbingjinguan 51 | (CT)_29/29_ | (AT)_13/16_ | (AG)_12/12_ | (AT)_7/7_ | (TA)_16/16_ | (CT)_19/23_ | (GA)_11/16_ | (CT)_20/20_ | T/T |
| 2336 Qinguang | (CT)_23/29_ | (AT)_13/16_ | (AG)_12/17_ | (AT)_7/13_ | (TA)_10/16_ | (CT)_19/23_ | (GA)_11/11_ | (CT)_14/14_ | T/C |
| Xingcheng 1518 | (CT)_23/29_ | (AT)_8/13_ | (AG)_12/12_ | (AT)_17/17_ | (TA)_16/16_ | (CT)_19/23_ | (GA)_11/16_ | (CT)_14/20_ | T/C |
| Xinhongxing | (CT)_17/23_ | (AT)_8/13_ | (AG)_12/12_ | (AT)_7/13_ | (TA)_16/16_ | (CT)_19/23_ | (GA)_16/16_ | (CT)_14/20_ | T/T |
| Zhanxuan 4 | (CT)_23/29_ | (AT)_8/13_ | (AG)_12/12_ | (AT)_13/13_ | (TA)_10/16_ | (CT)_19/23_ | (GA)_16/16_ | (CT)_14/20_ | T/C |
| Xingcheng 159 | (CT)_23/29_ | (AT)_8/13_ | (AG)_12/17_ | (AT)_7/7_ | (TA)_16/16_ | (CT)_19/23_ | (GA)_11/16_ | (CT)_20/20_ | T/T |
| Xinhong | (CT)_23/29_ | (AT)_8/13_ | (AG)_12/12_ | (AT)_7/13_ | (TA)_10/16_ | (CT)_19/23_ | (GA)_16/16_ | (CT)_20/20_ | T/T |
| Behene | (CT)_17/23_ | (AT)_13/16_ | (AG)_12/17_ | (AT)_13/13_ | (TA)_10/16_ | (CT)_19/23_ | (GA)_11/16_ | (CT)_14/20_ | T/T |
| Hadibolaite | (CT)_23/29_ | (AT)_8/13_ | (AG)_7/12_ | (AT)_7/13_ | (TA)_16/16_ | (CT)_19/23_ | (GA)_11/16_ | (CT)_14/14_ | T/C |
| Hongyindu | (CT)_17/23_ | (AT)_13/16_ | (AG)_12/12_ | (AT)_7/13_ | (TA)_10/16_ | (CT)_19/23_ | (GA)_16/16_ | (CT)_20/20_ | T/C |
| Zhuoai 1 | (CT)_23/29_ | (AT)_16/16_ | (AG)_12/12_ | (AT)_7/13_ | (TA)_16/16_ | (CT)_19/23_ | (GA)_16/16_ | (CT)_20/20_ | T/C |
| Chaohong | (CT)_23/29_ | (AT)_8/13_ | (AG)_7/7_ | (AT)_7/7_ | (TA)_16/16_ | (CT)_19/23_ | (GA)_11/16_ | (CT)_20/20_ | T/T |
| Zhanxuan 14 | (CT)_23/23_ | (AT)_13/16_ | (AG)_12/12_ | (AT)_13/13_ | (TA)_16/16_ | (CT)_19/23_ | (GA)_11/16_ | (CT)_20/20_ | T/C |
| Nanchengaijinguan | (CT)_23/29_ | (AT)_13/16_ | (AG)_12/12_ | (AT)_13/13_ | (TA)_16/16_ | (CT)_23/23_ | (GA)_16/16_ | (CT)_20/20_ | T/C |
| Ruiguang | (CT)_23/23_ | (AT)_13/16_ | (AG)_12/12_ | (AT)_7/17_ | (TA)_16/16_ | (CT)_19/23_ | (GA)_16/16_ | (CT)_14/14_ | T/C |
| Xinwojin | (CT)_23/29_ | (AT)_8/13_ | (AG)_7/12_ | (AT)_7/7_ | (TA)_10/16_ | (CT)_19/23_ | (GA)_16/16_ | (CT)_14/14_ | T/T |
| Hongxiezi | (CT)_23/29_ | (AT)_13/16_ | (AG)_12/17_ | (AT)_17/17_ | (TA)_10/16_ | (CT)_19/23_ | (GA)_11/16_ | (CT)_20/20_ | T/T |
| Dajin | (CT)_23/29_ | (AT)_13/16_ | (AG)_7/12_ | (AT)_7/7_ | (TA)_10/16_ | (CT)_19/23_ | (GA)_11/16_ | (CT)_14/20_ | T/C |
| Qinguan | (CT)_23/29_ | (AT)_13/13_ | (AG)_12/12_ | (AT)_7/13_ | (TA)_10/16_ | (CT)_19/23_ | (GA)_11/16_ | (CT)_14/14_ | T/T |
| Bankeluofute | (CT)_23/29_ | (AT)_16/16_ | (AG)_12/12_ | (AT)_17/17_ | (TA)_10/16_ | (CT)_23/23_ | (GA)_16/16_ | (CT)_20/20_ | T/T |
| Huangjin | (CT)_23/29_ | (AT)_13/16_ | (AG)_12/12_ | (AT)_7/13_ | (TA)_16/16_ | (CT)_19/23_ | (GA)_16/16_ | (CT)_20/20_ | T/C |
| Xingcheng 174 | (CT)_23/29_ | (AT)_13/16_ | (AG)_12/12_ | (AT)_7/7_ | (TA)_16/16_ | (CT)_19/23_ | (GA)_16/16_ | (CT)_20/20_ | T/T |
| 18688 | (CT)_23/29_ | (AT)_13/16_ | (AG)_7/7_ | (AT)_7/7_ | (TA)_16/16_ | (CT)_19/23_ | (GA)_16/16_ | (CT)_20/20_ | T/C |
| Huangpi | (CT)_23/29_ | (AT)_13/16_ | (AG)_7/12_ | (AT)_7/7_ | (TA)_16/16_ | (CT)_19/23_ | (GA)_11/16_ | (CT)_20/20_ | T/C |
| Rizhiwan | (CT)_23/29_ | (AT)_8/13_ | (AG)_12/12_ | (AT)_17/17_ | (TA)_10/10_ | (CT)_19/23_ | (GA)_16/16_ | (CT)_14/14_ | T/T |
| Yingjin | (CT)_23/29_ | (AT)_8/13_ | (AG)_12/17_ | (AT)_17/17_ | (TA)_10/16_ | (CT)_23/23_ | (GA)_16/16_ | (CT)_20/20_ | T/T |
| Fenghuangnuanhaitangguo | (CT)_29/29_ | (AT)_13/16_ | (AG)_12/12_ | (AT)_7/13_ | (TA)_10/16_ | (CT)_19/23_ | (GA)_16/16_ | (CT)_20/20_ | T/T |
| Jie 9 | (CT)_23/23_ | (AT)_8/13_ | (AG)_7/7_ | (AT)_7/13_ | (TA)_10/16_ | (CT)_23/23_ | (GA)_16/16_ | (CT)_14/14_ | T/C |
| Qingguan | (CT)_17/23_ | (AT)_13/13_ | (AG)_12/12_ | (AT)_7/13_ | (TA)_16/16_ | (CT)_23/26_ | (GA)_11/11_ | (CT)_14/20_ | T/T |
| Daguoguang | (CT)_17/23_ | (AT)_8/13_ | (AG)_12/12_ | (AT)_13/13_ | (TA)_10/10_ | (CT)_23/23_ | (GA)_11/11_ | (CT)_20/20_ | T/T |
| Chilong | (CT)_17/17_ | (AT)_13/16_ | (AG)_7/12_ | (AT)_7/13_ | (TA)_16/16_ | (CT)_23/26_ | (GA)_16/16_ | (CT)_14/20_ | T/C |
| Xingcheng192 | (CT)_17/23_ | (AT)_13/16_ | (AG)_7/12_ | (AT)_7/13_ | (TA)_10/16_ | (CT)_23/23_ | (GA)_16/16_ | (CT)_14/20_ | T/C |
| Qingxiangjiao | (CT)_23/29_ | (AT)_13/16_ | (AG)_12/12_ | (AT)_7/13_ | (TA)_16/16_ | (CT)_23/26_ | (GA)_16/16_ | (CT)_14/20_ | T/T |
| Hesefengli | (CT)_29/29_ | (AT)_8/8_ | (AG)_12/12_ | (AT)_7/13_ | (TA)_16/16_ | (CT)_23/26_ | (GA)_11/16_ | (CT)_14/20_ | T/T |
| Gudeboge | (CT)_23/29_ | (AT)_13/16_ | (AG)_7/12_ | (AT)_17/17_ | (TA)_10/16_ | (CT)_19/19_ | (GA)_11/16_ | (CT)_14/20_ | T/C |
| Xingcheng 1211 | (CT)_23/29_ | (AT)_8/13_ | (AG)_7/12_ | (AT)_7/13_ | (TA)_16/16_ | (CT)_19/19_ | (GA)_11/16_ | (CT)_20/20_ | T/T |
| Ziyu | (CT)_23/29_ | (AT)_8/13_ | (AG)_12/12_ | (AT)_13/13_ | (TA)_16/16_ | (CT)_23/23_ | (GA)_16/16_ | (CT)_20/20_ | T/C |
| Fuhuapi | (CT)_17/23_ | (AT)_13/16_ | (AG)_12/12_ | (AT)_13/13_ | (TA)_10/16_ | (CT)_23/26_ | (GA)_16/16_ | (CT)_20/20_ | T/C |
| Tianhongyu | (CT)_23/23_ | (AT)_13/16_ | (AG)_12/12_ | (AT)_7/13_ | (TA)_16/16_ | (CT)_23/26_ | (GA)_11/16_ | (CT)_14/20_ | T/C |
| Kunmasi | (CT)_17/17_ | (AT)_13/16_ | (AG)_7/12_ | (AT)_7/13_ | (TA)_16/16_ | (CT)_23/23_ | (GA)_11/16_ | (CT)_14/14_ | T/C |
| Cuiyu | (CT)_23/29_ | (AT)_8/13_ | (AG)_7/17_ | (AT)_7/13_ | (TA)_16/16_ | (CT)_19/19_ | (GA)_11/16_ | (CT)_14/20_ | T/C |
| 60-15-30 | (CT)_23/29_ | (AT)_13/16_ | (AG)_12/12_ | (AT)_17/17_ | (TA)_10/16_ | (CT)_23/26_ | (GA)_11/16_ | (CT)_14/20_ | T/C |
| Xinhongyu | (CT)_23/23_ | (AT)_13/16_ | (AG)_7/12_ | (AT)_13/13_ | (TA)_10/16_ | (CT)_19/23_ | (GA)_11/16_ | (CT)_20/20_ | T/T |
| Kelapu | (CT)_23/23_ | (AT)_13/16_ | (AG)_12/17_ | (AT)_17/17_ | (TA)_10/16_ | (CT)_23/26_ | (GA)_16/16_ | (CT)_20/20_ | T/T |
| Yinggelan | (CT)_23/29_ | (AT)_13/16_ | (AG)_12/17_ | (AT)_7/13_ | (TA)_16/16_ | (CT)_23/23_ | (GA)_16/16_ | (CT)_14/20_ | T/T |
| Qiujin | (CT)_23/23_ | (AT)_13/16_ | (AG)_12/12_ | (AT)_7/13_ | (TA)_16/16_ | (CT)_23/26_ | (GA)_11/16_ | (CT)_14/20_ | T/T |
| Yidoujin | (CT)_23/23_ | (AT)_8/13_ | (AG)_12/17_ | (AT)_7/7_ | (TA)_16/16_ | (CT)_23/23_ | (GA)_11/16_ | (CT)_20/20_ | T/C |
| Lanfengwang | (CT)_17/17_ | (AT)_13/13_ | (AG)_12/12_ | (AT)_13/13_ | (TA)_10/16_ | (CT)_19/19_ | (GA)_16/16_ | (CT)_20/20_ | T/C |
| Zhumaliya | (CT)_23/23_ | (AT)_13/16_ | (AG)_12/12_ | (AT)_13/17_ | (TA)_10/16_ | (CT)_19/23_ | (GA)_11/16_ | (CT)_14/14_ | T/T |
| Weiqinni | (CT)_23/29_ | (AT)_16/16_ | (AG)_7/12_ | (AT)_17/17_ | (TA)_10/10_ | (CT)_23/26_ | (GA)_11/16_ | (CT)_14/20_ | T/C |
| Xingcheng 2116 | (CT)_23/29_ | (AT)_13/16_ | (AG)_12/12_ | (AT)_17/17_ | (TA)_10/16_ | (CT)_19/19_ | (GA)_11/16_ | (CT)_20/20_ | T/T |
| Xingcheng 2117 | (CT)_29/29_ | (AT)_13/16_ | (AG)_12/12_ | (AT)_7/13_ | (TA)_16/16_ | (CT)_19/19_ | (GA)_11/16_ | (CT)_14/14_ | T/C |
| Jieba | (CT)_23/23_ | (AT)_8/13_ | (AG)_12/12_ | (AT)_17/17_ | (TA)_10/16_ | (CT)_23/23_ | (GA)_16/16_ | (CT)_20/20_ | T/T |
| Xingping | (CT)_17/23_ | (AT)_16/16_ | (AG)_12/12_ | (AT)_13/13_ | (TA)_10/16_ | (CT)_23/26_ | (GA)_16/16_ | (CT)_20/20_ | T/C |
| Beierpusi | (CT)_29/29_ | (AT)_8/13_ | (AG)_12/12_ | (AT)_7/13_ | (TA)_16/16_ | (CT)_23/26_ | (GA)_11/11_ | (CT)_14/14_ | T/C |
| Taipingyangmeigui | (CT)_17/23_ | (AT)_8/13_ | (AG)_12/12_ | (AT)_7/7_ | (TA)_10/16_ | (CT)_19/19_ | (GA)_11/16_ | (CT)_14/14_ | T/C |
| Duanzhiluao | (CT)_17/23_ | (AT)_8/13_ | (AG)_12/17_ | (AT)_17/17_ | (TA)_10/16_ | (CT)_19/19_ | (GA)_16/16_ | (CT)_20/20_ | T/T |
| Beidang | (CT)_23/29_ | (AT)_13/16_ | (AG)_12/12_ | (AT)_13/17_ | (TA)_16/16_ | (CT)_19/23_ | (GA)_11/16_ | (CT)_20/20_ | T/C |
| Dabinai | (CT)_17/23_ | (AT)_16/16_ | (AG)_7/12_ | (AT)_7/13_ | (TA)_10/10_ | (CT)_19/19_ | (GA)_11/16_ | (CT)_14/20_ | T/C |
| Liberty | (CT)_23/29_ | (AT)_13/16_ | (AG)_12/12_ | (AT)_13/13_ | (TA)_10/16_ | (CT)_19/19_ | (GA)_16/16_ | (CT)_20/20_ | T/T |
| Meixiang | (CT)_17/17_ | (AT)_13/16_ | (AG)_7/12_ | (AT)_17/17_ | (TA)_10/16_ | (CT)_23/26_ | (GA)_11/16_ | (CT)_20/20_ | T/C |
| Onieffnin | (CT)_23/29_ | (AT)_8/13_ | (AG)_12/12_ | (AT)_7/13_ | (TA)_10/16_ | (CT)_23/26_ | (GA)_11/16_ | (CT)_14/20_ | T/T |
| Meiguihong | (CT)_17/23_ | (AT)_8/13_ | (AG)_7/12_ | (AT)_7/13_ | (TA)_10/16_ | (CT)_23/26_ | (GA)_11/16_ | (CT)_20/20_ | T/C |
| Generos | (CT)_29/29_ | (AT)_16/16_ | (AG)_12/12_ | (AT)_7/13_ | (TA)_10/16_ | (CT)_23/26_ | (GA)_11/11_ | (CT)_14/20_ | T/T |
| Hahong | (CT)_23/23_ | (AT)_8/13_ | (AG)_7/12_ | (AT)_7/17_ | (TA)_10/16_ | (CT)_19/19_ | (GA)_11/16_ | (CT)_20/20_ | T/C |
| Xingcheng 231 | (CT)_23/23_ | (AT)_13/16_ | (AG)_12/12_ | (AT)_13/13_ | (TA)_16/16_ | (CT)_19/19_ | (GA)_11/16_ | (CT)_20/20_ | T/T |
| Xingcheng2310 | (CT)_17/29_ | (AT)_13/16_ | (AG)_7/12_ | (AT)_17/17_ | (TA)_16/16_ | (CT)_23/26_ | (GA)_16/16_ | (CT)_20/20_ | T/C |
| Kosttiq | (CT)_23/23_ | (AT)_13/16_ | (AG)_12/12_ | (AT)_13/13_ | (TA)_10/16_ | (CT)_19/23_ | (GA)_16/16_ | (CT)_20/20_ | T/T |
| Jinguang | (CT)_23/23_ | (AT)_13/16_ | (AG)_12/12_ | (AT)_17/17_ | (TA)_10/16_ | (CT)_19/23_ | (GA)_16/16_ | (CT)_14/20_ | T/C |
| Meina | (CT)_17/23_ | (AT)_13/16_ | (AG)_12/12_ | (AT)_17/17_ | (TA)_10/10_ | (CT)_23/26_ | (GA)_11/16_ | (CT)_14/20_ | T/C |
| B Jinguan | (CT)_23/29_ | (AT)_13/16_ | (AG)_12/12_ | (AT)_13/13_ | (TA)_16/16_ | (CT)_19/19_ | (GA)_11/16_ | (CT)_20/20_ | T/C |
| Holly（Hony） | (CT)_23/23_ | (AT)_13/16_ | (AG)_7/12_ | (AT)_17/17_ | (TA)_10/10_ | (CT)_19/23_ | (GA)_16/16_ | (CT)_14/20_ | T/C |
| Aozhou | (CT)_23/29_ | (AT)_8/13_ | (AG)_12/12_ | (AT)_7/13_ | (TA)_16/16_ | (CT)_19/23_ | (GA)_16/16_ | (CT)_14/20_ | T/C |
| Guoqin | (CT)_23/23_ | (AT)_13/16_ | (AG)_12/17_ | (AT)_13/17_ | (TA)_16/16_ | (CT)_19/23_ | (GA)_11/16_ | (CT)_20/20_ | T/T |
| Jiabukajinguang | (CT)_23/29_ | (AT)_8/13_ | (AG)_12/12_ | (AT)_13/13_ | (TA)_10/16_ | (CT)_19/19_ | (GA)_16/16_ | (CT)_20/20_ | T/C |
| Fengcunfushi | (CT)_23/29_ | (AT)_13/16_ | (AG)_7/12_ | (AT)_7/7_ | (TA)_10/16_ | (CT)_23/26_ | (GA)_11/16_ | (CT)_14/20_ | T/T |
| Qingming | (CT)_23/29_ | (AT)_13/16_ | (AG)_12/12_ | (AT)_7/13_ | (TA)_10/16_ | (CT)_23/26_ | (GA)_11/16_ | (CT)_14/14_ | T/C |
| Xingcheng 250 | (CT)_17/23_ | (AT)_13/16_ | (AG)_7/12_ | (AT)_7/13_ | (TA)_16/16_ | (CT)_19/19_ | (GA)_11/16_ | (CT)_14/20_ | T/C |
| Xingcheng 2510 | (CT)_23/29_ | (AT)_8/13_ | (AG)_7/12_ | (AT)_13/13_ | (TA)_16/16_ | (CT)_19/19_ | (GA)_16/16_ | (CT)_14/14_ | T/T |
| Ruidan | (CT)_17/17_ | (AT)_13/16_ | (AG)_12/12_ | (AT)_7/7_ | (TA)_10/16_ | (CT)_23/26_ | (GA)_11/16_ | (CT)_20/20_ | T/C |
| Aiwq | (CT)_23/29_ | (AT)_8/13_ | (AG)_12/17_ | (AT)_7/13_ | (TA)_16/16_ | (CT)_19/23_ | (GA)_16/16_ | (CT)_14/20_ | T/T |
| Micui | (CT)_23/29_ | (AT)_8/13_ | (AG)_12/17_ | (AT)_7/13_ | (TA)_10/16_ | (CT)_19/19_ | (GA)_16/16_ | (CT)_14/20_ | T/C |
| Youlixiang | (CT)_23/29_ | (AT)_8/13_ | (AG)_12/12_ | (AT)_13/13_ | (TA)_10/16_ | (CT)_19/23_ | (GA)_16/16_ | (CT)_14/20_ | T/C |
| Fuqiu | (CT)_17/17_ | (AT)_13/13_ | (AG)_7/12_ | (AT)_17/17_ | (TA)_10/16_ | (CT)_19/23_ | (GA)_16/16_ | (CT)_14/14_ | T/C |
| Zhongxin | (CT)_23/29_ | (AT)_8/13_ | (AG)_12/12_ | (AT)_7/13_ | (TA)_16/16_ | (CT)_23/26_ | (GA)_11/16_ | (CT)_14/20_ | T/C |
| Weixishengming | (CT)_29/29_ | (AT)_13/16_ | (AG)_7/12_ | (AT)_17/17_ | (TA)_10/16_ | (CT)_23/26_ | (GA)_11/16_ | (CT)_20/20_ | T/T |
| Qihu 1 | (CT)_23/23_ | (AT)_8/13_ | (AG)_7/12_ | (AT)_13/13_ | (TA)_10/16_ | (CT)_23/26_ | (GA)_11/16_ | (CT)_14/14_ | T/C |
| Szampion(Szamlion | (CT)_23/29_ | (AT)_13/16_ | (AG)_12/12_ | (AT)_7/7_ | (TA)_16/16_ | (CT)_19/19_ | (GA)_16/16_ | (CT)_20/20_ | T/T |
| Hongxue | (CT)_23/29_ | (AT)_13/13_ | (AG)_12/12_ | (AT)_7/7_ | (TA)_16/16_ | (CT)_19/23_ | (GA)_11/16_ | (CT)_20/20_ | T/T |
| Xingcheng 2614 | (CT)_29/29_ | (AT)_8/13_ | (AG)_12/12_ | (AT)_7/17_ | (TA)_10/16_ | (CT)_19/19_ | (GA)_16/16_ | (CT)_14/14_ | T/C |
| Jinaisheng | (CT)_23/23_ | (AT)_13/13_ | (AG)_7/17_ | (AT)_13/13_ | (TA)_10/16_ | (CT)_23/26_ | (GA)_11/16_ | (CT)_20/20_ | T/C |
| Gaoqiu | (CT)_29/29_ | (AT)_13/16_ | (AG)_17/17_ | (AT)_7/13_ | (TA)_16/16_ | (CT)_19/23_ | (GA)_11/11_ | (CT)_14/20_ | T/C |
| Changhong | (CT)_23/23_ | (AT)_13/16_ | (AG)_7/12_ | (AT)_7/7_ | (TA)_16/16_ | (CT)_19/23_ | (GA)_11/16_ | (CT)_14/20_ | T/T |
| Yanfu 1 | (CT)_23/23_ | (AT)_16/16_ | (AG)_7/12_ | (AT)_7/13_ | (TA)_16/16_ | (CT)_19/23_ | (GA)_11/16_ | (CT)_14/14_ | T/T |
| Danxia | (CT)_23/23_ | (AT)_13/16_ | (AG)_12/12_ | (AT)_7/13_ | (TA)_16/16_ | (CT)_19/23_ | (GA)_11/11_ | (CT)_14/14_ | T/T |
| Ruilianna | (CT)_23/29_ | (AT)_13/16_ | (AG)_17/17_ | (AT)_7/13_ | (TA)_10/16_ | (CT)_19/23_ | (GA)_16/16_ | (CT)_14/14_ | T/C |
| Ⅰ11－2 | (CT)_23/29_ | (AT)_13/16_ | (AG)_7/12_ | (AT)_13/13_ | (TA)_10/16_ | (CT)_19/19_ | (GA)_11/16_ | (CT)_20/20_ | T/C |
| Zaoshenghezhinuan | (CT)_23/29_ | (AT)_13/16_ | (AG)_12/12_ | (AT)_13/13_ | (TA)_10/16_ | (CT)_23/26_ | (GA)_11/11_ | (CT)_14/20_ | T/C |
| Hui | (CT)_17/17_ | (AT)_13/16_ | (AG)_7/7_ | (AT)_13/13_ | (TA)_16/16_ | (CT)_19/19_ | (GA)_16/16_ | (CT)_20/20_ | T/C |
| Miguo | (CT)_23/23_ | (AT)_13/16_ | (AG)_12/12_ | (AT)_17/17_ | (TA)_10/16_ | (CT)_19/23_ | (GA)_11/16_ | (CT)_20/20_ | T/C |
| Xinqiaonajin | (CT)_29/29_ | (AT)_8/13_ | (AG)_12/12_ | (AT)_7/13_ | (TA)_10/16_ | (CT)_19/23_ | (GA)_16/16_ | (CT)_20/20_ | T/T |
| K12 | (CT)_29/29_ | (AT)_13/16_ | (AG)_12/12_ | (AT)_17/17_ | (TA)_16/16_ | (CT)_19/23_ | (GA)_11/16_ | (CT)_20/20_ | T/T |
| K9 | (CT)_23/23_ | (AT)_13/16_ | (AG)_12/12_ | (AT)_7/13_ | (TA)_10/16_ | (CT)_19/23_ | (GA)_11/16_ | (CT)_14/20_ | T/C |
| Liuyuyabian | (CT)_23/29_ | (AT)_13/16_ | (AG)_17/17_ | (AT)_7/13_ | (TA)_16/16_ | (CT)_19/23_ | (GA)_11/16_ | (CT)_14/20_ | T/T |
| Suyisiliebo | (CT)_23/29_ | (AT)_13/16_ | (AG)_12/12_ | (AT)_17/17_ | (TA)_10/16_ | (CT)_19/23_ | (GA)_11/16_ | (CT)_14/20_ | T/C |
| Baosikupu | (CT)_23/23_ | (AT)_8/13_ | (AG)_7/7_ | (AT)_13/13_ | (TA)_10/16_ | (CT)_23/23_ | (GA)_11/16_ | (CT)_14/14_ | T/C |
| Liuyuyabian | (CT)_23/23_ | (AT)_8/16_ | (AG)_12/12_ | (AT)_13/13_ | (TA)_16/16_ | (CT)_19/19_ | (GA)_16/16_ | (CT)_20/20_ | T/T |
| Shiai | (CT)_23/23_ | (AT)_13/13_ | (AG)_7/12_ | (AT)_13/13_ | (TA)_10/16_ | (CT)_23/26_ | (GA)_16/16_ | (CT)_20/20_ | T/C |
| Xingcheng 220 | (CT)_17/23_ | (AT)_8/13_ | (AG)_12/17_ | (AT)_13/13_ | (TA)_16/16_ | (CT)_19/19_ | (GA)_11/16_ | (CT)_20/20_ | T/C |
| Shalatuoni | (CT)_23/29_ | (AT)_8/13_ | (AG)_12/12_ | (AT)_7/13_ | (TA)_10/10_ | (CT)_19/19_ | (GA)_11/16_ | (CT)_14/20_ | T/C |
| Xingcheng 300 | (CT)_17/23_ | (AT)_13/16_ | (AG)_12/17_ | (AT)_13/13_ | (TA)_16/16_ | (CT)_23/26_ | (GA)_11/16_ | (CT)_20/20_ | T/C |
| Lubi | (CT)_23/29_ | (AT)_13/16_ | (AG)_7/12_ | (AT)_7/13_ | (TA)_16/16_ | (CT)_19/23_ | (GA)_11/16_ | (CT)_20/20_ | T/T |
| Xingcheng 319 | (CT)_23/29_ | (AT)_8/13_ | (AG)_12/12_ | (AT)_7/13_ | (TA)_10/16_ | (CT)_19/23_ | (GA)_16/16_ | (CT)_20/20_ | T/C |
| Senmalan | (CT)_23/29_ | (AT)_8/16_ | (AG)_12/17_ | (AT)_7/7_ | (TA)_16/16_ | (CT)_19/23_ | (GA)_16/16_ | (CT)_20/20_ | T/T |
| Kuihua | (CT)_23/29_ | (AT)_8/13_ | (AG)_12/12_ | (AT)_7/13_ | (TA)_10/16_ | (CT)_19/23_ | (GA)_16/16_ | (CT)_14/20_ | T/C |
| Miqiulinjilian | (CT)_23/29_ | (AT)_13/16_ | (AG)_7/12_ | (AT)_17/17_ | (TA)_10/16_ | (CT)_19/19_ | (GA)_11/16_ | (CT)_14/20_ | T/C |
| Weisitabeila | (CT)_23/29_ | (AT)_8/13_ | (AG)_12/12_ | (AT)_7/13_ | (TA)_16/16_ | (CT)_19/23_ | (GA)_16/16_ | (CT)_20/20_ | T/C |
| 4F18 | (CT)_23/23_ | (AT)_8/13_ | (AG)_7/7_ | (AT)_7/17_ | (TA)_10/10_ | (CT)_19/23_ | (GA)_16/16_ | (CT)_14/14_ | T/C |
| Qiheduanjinguan | (CT)_23/29_ | (AT)_16/16_ | (AG)_12/12_ | (AT)_13/13_ | (TA)_16/16_ | (CT)_23/23_ | (GA)_11/16_ | (CT)_20/20_ | T/C |
| Xingcheng 423 | (CT)_29/29_ | (AT)_8/13_ | (AG)_7/12_ | (AT)_13/13_ | (TA)_16/16_ | (CT)_19/19_ | (GA)_11/16_ | (CT)_14/20_ | T/C |
| Zaoshengchi | (CT)_23/29_ | (AT)_13/16_ | (AG)_12/12_ | (AT)_7/13_ | (TA)_10/10_ | (CT)_23/26_ | (GA)_16/16_ | (CT)_20/20_ | T/T |
| Qiaonajin | (CT)_29/29_ | (AT)_16/16_ | (AG)_12/12_ | (AT)_13/13_ | (TA)_10/16_ | (CT)_19/23_ | (GA)_11/16_ | (CT)_14/14_ | T/C |
| Ayiwaniya | (CT)_23/29_ | (AT)_16/16_ | (AG)_17/17_ | (AT)_13/17_ | (TA)_16/16_ | (CT)_23/26_ | (GA)_16/16_ | (CT)_20/20_ | T/T |
| Fushan 5 | (CT)_29/29_ | (AT)_16/16_ | (AG)_12/12_ | (AT)_7/13_ | (TA)_10/10_ | (CT)_19/19_ | (GA)_16/16_ | (CT)_20/20_ | T/T |
| Xingcheng 52 | (CT)_23/23_ | (AT)_16/16_ | (AG)_12/12_ | (AT)_17/17_ | (TA)_10/10_ | (CT)_19/23_ | (GA)_16/16_ | (CT)_14/14_ | T/C |
| Mijin | (CT)_23/29_ | (AT)_8/8_ | (AG)_12/17_ | (AT)_7/17_ | (TA)_10/10_ | (CT)_19/23_ | (GA)_11/16_ | (CT)_20/20_ | T/C |
| Sitakeaijinguan | (CT)_23/29_ | (AT)_13/16_ | (AG)_12/12_ | (AT)_13/13_ | (TA)_10/16_ | (CT)_23/26_ | (GA)_16/16_ | (CT)_20/20_ | T/T |
| Enweierjinaisheng | (CT)_23/29_ | (AT)_13/16_ | (AG)_12/12_ | (AT)_17/17_ | (TA)_10/16_ | (CT)_23/23_ | (GA)_16/16_ | (CT)_20/20_ | T/C |
| Aihong | (CT)_23/23_ | (AT)_8/13_ | (AG)_7/12_ | (AT)_7/13_ | (TA)_16/16_ | (CT)_19/23_ | (GA)_11/11_ | (CT)_14/14_ | T/T |
| Qingdao 1 | (CT)_23/29_ | (AT)_8/13_ | (AG)_7/12_ | (AT)_7/13_ | (TA)_16/16_ | (CT)_19/19_ | (GA)_11/16_ | (CT)_14/14_ | T/C |
| Bianqiangzi 1 | (CT)_17/23_ | (AT)_8/13_ | (AG)_7/12_ | (AT)_7/7_ | (TA)_16/16_ | (CT)_23/26_ | (GA)_11/16_ | (CT)_14/14_ | T/T |
| Xinjiang327 | (CT)_23/29_ | (AT)_8/8_ | (AG)_7/12_ | (AT)_7/13_ | (TA)_10/16_ | (CT)_23/26_ | (GA)_11/16_ | (CT)_20/20_ | T/C |
| 132 | (CT)_17/23_ | (AT)_8/13_ | (AG)_7/12_ | (AT)_7/13_ | (TA)_16/16_ | (CT)_19/23_ | (GA)_11/16_ | (CT)_14/20_ | T/T |
| Meiduan 1 | (CT)_23/23_ | (AT)_8/13_ | (AG)_7/12_ | (AT)_17/17_ | (TA)_16/16_ | (CT)_19/23_ | (GA)_11/16_ | (CT)_14/20_ | T/T |
| De 14 | (CT)_23/29_ | (AT)_8/13_ | (AG)_12/12_ | (AT)_7/17_ | (TA)_10/16_ | (CT)_19/19_ | (GA)_16/16_ | (CT)_20/20_ | T/C |
| Qiujinxing | (CT)_29/29_ | (AT)_16/16_ | (AG)_12/17_ | (AT)_7/7_ | (TA)_16/16_ | (CT)_19/19_ | (GA)_11/16_ | (CT)_20/20_ | T/C |
| Kangtunduanzhi | (CT)_23/23_ | (AT)_8/8_ | (AG)_7/12_ | (AT)_7/13_ | (TA)_10/16_ | (CT)_19/23_ | (GA)_11/16_ | (CT)_20/20_ | T/C |
| Xingcheng 77 | (CT)_23/29_ | (AT)_8/13_ | (AG)_7/12_ | (AT)_7/7_ | (TA)_10/16_ | (CT)_19/23_ | (GA)_11/16_ | (CT)_20/20_ | T/T |
| Xingcheng 79 | (CT)_23/23_ | (AT)_13/16_ | (AG)_12/12_ | (AT)_7/7_ | (TA)_16/16_ | (CT)_19/23_ | (GA)_11/11_ | (CT)_20/20_ | T/C |
| Zakeliebieer | (CT)_17/23_ | (AT)_13/13_ | (AG)_12/12_ | (AT)_17/17_ | (TA)_16/16_ | (CT)_23/23_ | (GA)_11/16_ | (CT)_20/20_ | T/C |
| Mesiketouming | (CT)_17/23_ | (AT)_8/13_ | (AG)_7/12_ | (AT)_13/13_ | (TA)_16/16_ | (CT)_19/19_ | (GA)_11/11_ | (CT)_20/20_ | T/C |
| Xiteshisheng | (CT)_29/29_ | (AT)_8/13_ | (AG)_12/12_ | (AT)_13/13_ | (TA)_10/16_ | (CT)_23/26_ | (GA)_11/16_ | (CT)_20/20_ | T/C |
| Zakeliebieer | (CT)_17/23_ | (AT)_8/13_ | (AG)_12/12_ | (AT)_7/13_ | (TA)_10/10_ | (CT)_23/26_ | (GA)_16/16_ | (CT)_20/20_ | T/T |
| Magu | (CT)_23/29_ | (AT)_8/13_ | (AG)_7/12_ | (AT)_17/17_ | (TA)_10/16_ | (CT)_19/23_ | (GA)_11/16_ | (CT)_20/20_ | T/T |
| Ximengfei | (CT)_17/23_ | (AT)_8/13_ | (AG)_12/12_ | (AT)_7/13_ | (TA)_10/16_ | (CT)_19/23_ | (GA)_16/16_ | (CT)_14/14_ | T/T |
| De 2 | (CT)_23/29_ | (AT)_8/13_ | (AG)_12/12_ | (AT)_13/13_ | (TA)_16/16_ | (CT)_19/23_ | (GA)_16/16_ | (CT)_20/20_ | T/T |
| Yeweilin | (CT)_17/23_ | (AT)_8/16_ | (AG)_12/17_ | (AT)_17/17_ | (TA)_10/16_ | (CT)_19/19_ | (GA)_11/16_ | (CT)_20/20_ | T/T |
| Situonuowei | (CT)_23/29_ | (AT)_13/16_ | (AG)_12/12_ | (AT)_7/13_ | (TA)_16/16_ | (CT)_19/23_ | (GA)_11/16_ | (CT)_20/20_ | T/C |
| Xingcheng 923 | (CT)_23/29_ | (AT)_13/16_ | (AG)_12/12_ | (AT)_13/13_ | (TA)_16/16_ | (CT)_19/23_ | (GA)_11/16_ | (CT)_20/20_ | T/C |
| Xingcheng 93 | (CT)_17/17_ | (AT)_8/8_ | (AG)_12/17_ | (AT)_7/13_ | (TA)_16/16_ | (CT)_23/26_ | (GA)_11/16_ | (CT)_20/20_ | T/T |
| Keluodeng | (CT)_23/29_ | (AT)_8/13_ | (AG)_12/12_ | (AT)_13/17_ | (TA)_16/16_ | (CT)_19/23_ | (GA)_16/16_ | (CT)_14/20_ | T/T |
